# Diversity in parasitic nematode genomes: the microRNAs of *Brugia pahangi *and *Haemonchus contortus *are largely novel

**DOI:** 10.1186/1471-2164-13-4

**Published:** 2012-01-04

**Authors:** Alan D Winter, William Weir, Martin Hunt, Matthew Berriman, John S Gilleard, Eileen Devaney, Collette Britton

**Affiliations:** 1 Institute of Infection, Immunity and Inflammation; College of Medical, Veterinary and Life Sciences; University of Glasgow, Garscube Estate, Bearsden Road, Glasgow, G61 1QH, UK; 2 Wellcome Trust Sanger Institute, Hinxton, Cambridge, CB10 1SA, UK; 3 Department of Comparative Biology and Experimental Medicine, Faculty of Veterinary Medicine, University of Calgary, Calgary, Alberta, T2N 4N1, Canada

## Abstract

**Background:**

MicroRNAs (miRNAs) play key roles in regulating post-transcriptional gene expression and are essential for development in the free-living nematode *Caenorhabditis elegans *and in higher organisms. Whether microRNAs are involved in regulating developmental programs of parasitic nematodes is currently unknown. Here we describe the the miRNA repertoire of two important parasitic nematodes as an essential first step in addressing this question.

**Results:**

The small RNAs from larval and adult stages of two parasitic species, *Brugia pahangi *and *Haemonchus contortus*, were identified using deep-sequencing and bioinformatic approaches. Comparative analysis to known miRNA sequences reveals that the majority of these miRNAs are novel. Some novel miRNAs are abundantly expressed and display developmental regulation, suggesting important functional roles. Despite the lack of conservation in the miRNA repertoire, genomic positioning of certain miRNAs within or close to specific coding genes is remarkably conserved across diverse species, indicating selection for these associations. Endogenous small-interfering RNAs and Piwi-interacting (pi)RNAs, which regulate gene and transposon expression, were also identified. piRNAs are expressed in adult stage *H. contortus*, supporting a conserved role in germline maintenance in some parasitic nematodes.

**Conclusions:**

This in-depth comparative analysis of nematode miRNAs reveals the high level of divergence across species and identifies novel sequences potentially involved in development. Expression of novel miRNAs may reflect adaptations to different environments and lifestyles. Our findings provide a detailed foundation for further study of the evolution and function of miRNAs within nematodes and for identifying potential targets for intervention.

## Background

microRNAs (miRNAs) are small, non-coding RNAs that play key roles in regulating gene expression in animals, plants and viruses. The processing of miRNAs from long primary transcripts to their mature functional form by Drosha and Dicer RNase III enzymes has been reviewed recently [1]. The mature ~22-nucleotide single-stranded molecule is incorporated into an RNA-induced silencing complex (RISC), directing it to mRNA targets resulting in their translational repression or destabilisation [2]. In animals, miRNA target sites most often show partial sequence complementarity and, although best characterised in 3'UTRs, can occur anywhere in the gene [3,4]. Accumulating evidence suggests that the majority of mRNAs may be targets of miRNA regulation [5], providing an increased level of complexity to global gene control.

miRNAs show temporally controlled and cell-specific patterns of expression [6-8], with the first level of regulation being transcriptional. However, the importance of post-transcriptional regulation is becoming increasingly apparent [9]. In mammals, miRNAs have been shown to regulate diverse and important processes such as B-cell differentiation [10], adipocyte differentiation [11], cardiogenesis [12], insulin secretion [13], antiviral defence [14], and the development of cancer [15,16].

Genetic analysis in the free-living nematode *Caenorhabditis elegans *led to the discovery of the first miRNA, *lin-4*, which controls hypodermal cell fate decisions during early larval development [17,18]. Similarly, *C. elegans let-7 *and the related miRNAs*, mir-48*, *mir-84 *and *mir-241*, also function to regulate the timing of developmental events [19,20]. A cell-specific role has been defined for *C. elegans lys-6 *and *mir-273*, which act sequentially to control the laterality of chemosensory neurons [21,22]. However, in most cases, mutation of other *C. elegans *miRNAs, either individually [23] or by combined mutation of related sequences [24], results in no observable effect on development or viability. Exceptions are the early lethality phenotypes resulting from both the combined loss of *mir-35*-*mir-42 *[24] and of *mir-51-mir-56*, [24,25], as well as the movement and body size defect resulting from combined mutation of *mir-58*, *-80*, *-81 *and *-82 *[24]. Importantly, miRNAs which are unrelated in sequence may function in concert as demonstrated by the phenotypes of individual miRNA mutants, which are revealed only in genetic backgrounds where miRNA levels are globally reduced [26]. Additionally, function may only become apparent after detailed analysis, as demonstrated for *C. elegans mir-1*, which regulates synaptic signalling at neuromuscular junctions and influences sensitivity to levamisole [27].

Parasitic nematodes of medical and veterinary importance have body plans and developmental programs similar to *C. elegans*. Whether development of parasitic species is regulated by microRNAs, as occurs in *C. elegans*, is currently unknown. As an essential first step to address this question we have undertaken a comprehensive analysis of miRNAs in two parasitic nematodes: *Brugia pahangi*, a mosquito-borne parasite closely related to *B. malayi *and *Wuchereria bancrofti*, which are the causative agents of lymphatic filariasis, a condition affecting around 120 million people in 73 countries worldwide [28,29]; and *Haemonchus contortus*, a prevalent blood-feeding gastrointestinal parasite of ruminants causing substantial losses to livestock production. *H. contortus *is relatively closely related to *C. elegans*, with both belonging to nematode clade V, while *Brugia *spp. (clade III) are more distantly related [30]. Importantly, extensive genome sequence information is available for *B. malayi *[31] and *H. contortus *[32], allowing identification of miRNAs by deep-sequencing and bioinformatic approaches. Here we perform a genome-wide discovery of small RNAs in *B. pahangi *and *H. contortus *and compare the repertoire of miRNAs to those in well-studied species, particularly *C. elegans*. Surprisingly, despite the close phylogenetic relationship, particularly between *C. elegans *and *H. contortus*, we find that the majority of miRNAs from the parasitic nematodes are novel, perhaps reflecting adaptations to parasitism.

miRNAs are members of a larger group of small silencing non-coding RNAs, including endogenous small-interfering (endo-si)RNAs and Piwi-interacting (pi)RNAs, which are classified on the basis of their biogenesis and the proteins with which they associate in the RISC complex [1,33]. Here, our discovery of endo-siRNAs in both parasite species and piRNAs in *H. contortus *provides an in-depth picture of the small RNA silencing pathways operating in gene regulation and genome stability in these organisms. Identification of these molecules will facilitate detailed study of their function and may ultimately provide novel targets for parasitic nematode control.

## Results

### Small RNA library sequence analysis

Deep sequencing has been used effectively for small RNA discovery and expression profiling in diverse organisms [34-36]. *B. pahangi *is a sister species to the human-infective filarial nematode *B. malayi *and shows a high level of conservation of gene sequences, including 18S rRNAs [37], mRNAs [38], and their *Wolbachia *endosymbionts [39]. The complete lifecycle of *B. pahangi *is maintained in our laboratory, allowing access to all developmental stages. To enable identification of small RNAs we exploited the almost complete and annotated genome sequence data for *B. malayi *[31] and mapped *B. pahangi *sequences to this genome. Sequences identified in this manner were considered common to *B. pahangi *and *B. malayi *and, as such, are referred to as *Brugia *small RNAs. The *B. malayi *genome data represents 88 Mb of assembled sequence, mostly in scaffolds (N50 = 93,771 bp), with an estimated genome size of 90-95 Mb [31]. The *H. contortus *genome sequencing project is ongoing with 900 Mb of sequence data estimated to represent 2-3 fold genome coverage (JSG and MB, unpublished data). The majority of sequences have been assembled into contigs (N50 = 2,370 bp) and supercontigs (N50 = 9,901 bp) with annotation presently unpublished. In this study we carried out Illumina deep sequencing [40] of small RNA libraries prepared from infective third stage larvae (L3) and mixed-sex adult stages of *B. pahangi *and *H. contortus *which produced between 5.2 and 13.2 million raw reads (Table 1). Only reads with perfect matches to the genome data were retained, which identified between 268,717 and 748,568 unique mapped sequences for each library, representing 3.6-8.2 million total mapped reads (Table 1). Short read sequence data from this study have been submitted to the NCBI Gene Expression Omnibus (GEO) under series accession number GSE34539.

**Table 1:** Summary of small RNA library sequencing

	Library			
	***B. pahangi *L3**	***B. pahangi *adult**	***H. contortus *L3**	***H. contortus *adult**

**Raw reads**	13242069	13164896	5198711	12226463
**Unique mapped sequences**	268717	424849	244003	748568
**Total mapped reads**	3853899	8196608	3644275	5666474
**Percentage mapped**	29.1%	62.3%	70.1%	46.3%
**tRNA**	164815	314999	44469	161537
**rRNA**	463942	567695	1774431	1826279
**Repeat^a^**	22	305	-	-
**miRNA (mature)**	2359229	5729861	1193015	1412260
**miRNA (star)**	16560	115880	2862	8811
**cds/exon (sense)**	263828	294245	227319	266308
**cds/exon (antisense)**	140829	391339	283917	271624
**piRNA^b^**	(44)	(49)	(15)	1296
**Other**	444630	782235	118247	1718359

^a ^Not analysed for *H. contortus*.

^b ^Only the *H. contortus *adult sequences are likely to represent genuine piRNAs.

**Figure 1:**
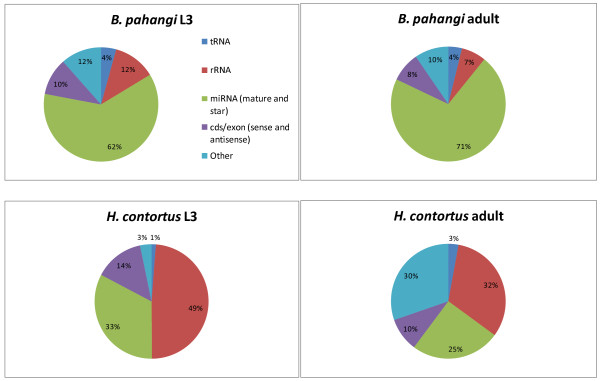
**Small RNA library composition**. The proportion of reads representing different classes of RNAs, relative to the total number of mapped reads for each small RNA library, is shown. Reads matching repeats and possible piRNAs are not represented due to their low relative abundance.

Computational approaches were then used to classify sequences as miRNA, siRNA or piRNA and identify reads derived from rRNAs and tRNAs. The proportion of reads assigned to each RNA class for the L3 and adult stage libraries of both species is shown in Figure 1 and in more detail in Table 1. Examining the composition of the libraries showed that for *B. pahangi*, miRNA-derived reads were the most abundant in both stages (62-71%). The miRNA contribution was lower in both *H. contortus *libraries (25-33%), which was a consequence of a higher percentage of rRNA sequences. Variable levels of rRNA sequences in small RNA libraries have been described previously [41,42].

### miRNA identification from sequencing data

Two miRNA prediction programs, miRDeep [43] and MIREAP [44] were used to identify miRNAs from the small RNA deep-sequencing data, both of which have been used previously for worm (nematode and platyhelminth) miRNA identification [41-43,45]. These software utilise algorithms which score sequences on the likelihood of representing true miRNAs by folding excised genomic regions flanking mapped reads and scoring these structures based on their free-energy, similarity to miRNA hairpins, and evidence of processing by Drosha and Dicer. Neither program relies on homology to known miRNA sequences for identification thereby enabling novel miRNA discovery.

Preliminary analysis of the output from both programs suggested that reliance on a single program could result in failure to identify some miRNAs. Comparing the *B. pahangi *output for both programs with the 32 known sequences identified previously for *B. malayi *[46] showed that while 28 sequences were successfully identified, four were found only by miRDeep and three, including *let-7*, were found only by MIREAP. Therefore, to reduce the likelihood of failing to identify genuine miRNAs, the prediction outputs from both programs (minimum free energy, MFE, ≤-25 kcal/mol) were combined. These combined output data was subsequently processed to identify identical, or near identical, sequences mapping to different genomic locations (also see Supplemental Results in Additional File 1), and to find sequences common to both developmental stages. 154 *B. pahangi *and 384 *H. contortus *sequences identified by this process were then filtered to remove those matching known rRNA, tRNA, transposon or repeat sequences resulting in 149 and 367 sequences, respectively. We then further filtered these on the basis of star strand identification (the minor ~22 nucleotide product, partially complementary to the mature, or major, product), read counts and a further hairpin classifier, CIDmiRNA [47], to reduce the chance of false miRNA annotation and produce a final high confidence set. For *B. pahangi*, this produced 125 precursor sequences representing 123 miRNA loci, encoding 99 unique mature sequences, and 81 unique star sequences. For *H. contortus*, the same process identified 180 precursor sequences representing 175 loci, encoding 180 unique mature sequences and 128 unique star sequences (Table 2). Full details of the final miRNAs are given in Additional File 2. miRNA sequences have been submitted the EMBL Nucleotide Sequence Database (accession numbers HE617336-HE617661) and to miRBase, the miRNA database. The final numbers of precursors identified by each program or combinations of programs are shown in Additional File 1, Table S1. Consistent with previous studies [48], most miRNAs identified in both parasitic nematodes were 21-24 nucleotides in length (Additional File 1, Figure S1) with a preference for a 5' uracil (45% for *B. pahangi *and 52% for *H. contortus*). We also find evidence for a small number of additional miRNAs in the unmapped sequence data (see Supplemental Results in Additional File 1).

**Table 2:** miRNA numbers discovered by deep sequencing and bioinformatic approaches

	*Brugia*				*H. contortus*			
	**Sequences**	**Loci**	**Mature**	**Star**	**Sequences**	**Loci**	**Mature**	**Star**

**Sequencing**	125	123	99	81	180	175	180	128
**Bioinformatic**	9	9	5	-	12	12	12	-
**Total**	134	132	104	81	192	187	192	128

### Homology-based computational miRNA discovery

Sequencing approaches to miRNA discovery can fail to identify those that are expressed either at low levels, in limited cell types or during developmental stages not sampled. A computational homology-based discovery approach was therefore employed to determine if additional miRNAs could be identified in the available *B. malayi *and *H. contortus *genome data. The mature sequences of worm miRNAs present in miRBase (release 15) were used as BLAST [49] queries against *B. malayi *and *H. contortus *genome sequences. The flanking genomic regions from BLAST hits were excised in two orientations and these candidate precursor sequences passed through RNAfold [50] and CIDmiRNA [47] filtering steps, followed by manual examination (using criteria described in Supplemental Methods in Additional File 1). As well as identifying 22 *Brugia *and 25 *H. contortus *miRNAs that we had already found by deep-sequencing, this bioinformatics approach identified an additional 42 *Brugia *and 78 *H. contortus *sequences. Final high confidence sets of five *Brugia *and 12 *H. contortus *sequences (Table 2 and Additional File 3) were then generated by retaining only those sequences that passed specific criteria (as described in Supplemental Methods). Therefore through the complementary discovery approaches of deep-sequencing and bioinformatics we identify a total of 132 *Brugia *miRNA loci encoding 104 unique mature sequences, and for *H. contortus *187 loci encoding 192 unique mature sequences (Table 2). The small number of additional homology-identified miRNAs supports the efficiency of the deep-sequencing approach and also suggests that the low number of conserved miRNAs found (see below) is an accurate picture of the miRNA populations in these parasite stages.

A comparison of the *Brugia *miRNAs discovered here with the 32 *B. malayi *miRNAs identified previously [46] showed that all apart from *mir-72*, *-124 *and *-153 *were identified in our analysis. Reads identical to the *mir-72 *mature sequence were present in the *B. pahangi *L3 and adult data but were not classified as miRNA by the programs used, possibly as a result of the unusual precursor structure. *mir-124 *and *mir-153 *were not identified here as they were originally found from the sequence trace archive [46] with the full precursor sequences not being contained in the reference genome sequence used here. In contrast to the previous annotation for *B. malayi mir-36a *and *Bma-mir-36b*, we found the major products from these loci were derived from the opposite arms of the hairpins, which were > 4-fold more abundant and were represented by > 50,000 reads. This finding could either represent differences between *Brugia *species and be an example of arm switching (see later section), or represent more accurate annotation due to the depth of our sequencing data.

### High abundance miRNAs

**Figure 2:**
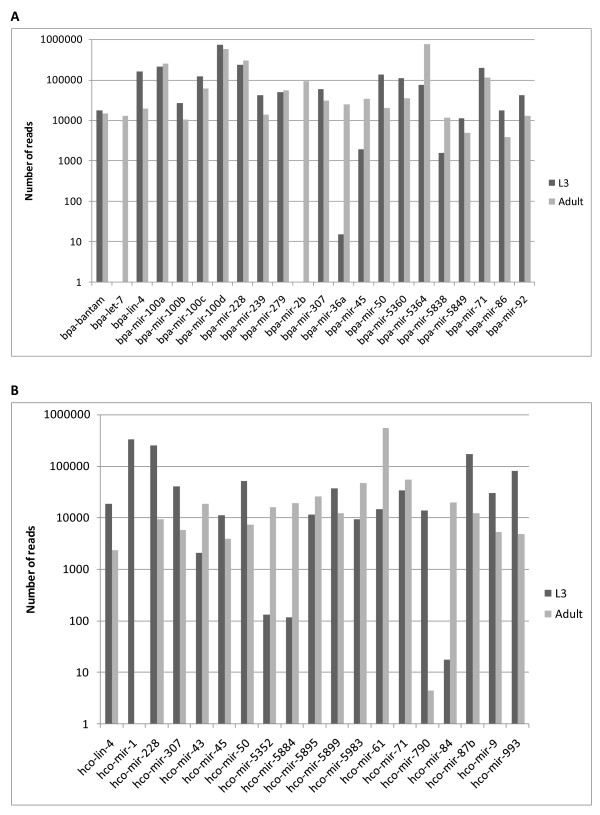
**High abundance miRNAs**. miRNAs found at high abundance (> 10,000 reads) in at least one developmental stage. Read numbers are normalised and illustrated on a log scale with the read numbers indicated. A) 22 abundant *B. pahangi *miRNAs, B) 19 abundant *H. contortus *miRNAs.

Previous studies have used the number of sequence reads of a particular miRNA from deep-sequencing as an indication of molecular abundance [42]. Therefore to gain an approximate measure of expression we examined the read numbers for each unique miRNA in the L3 and adult stages of *B. pahangi *and *H. contortus*, after normalisation of the data to the total number of reads mapping to the genome for each library. 22 *B. pahangi *and 19 *H. contortus *miRNAs were found at high abundance (> 10,000 reads), either in the L3 stage, adult stage, or both (Figure 2A and 2B). In keeping with data from other organisms, many of these are evolutionarily conserved (see below). Of the highly expressed miRNAs common to both parasites, five (*lin-4*, *mir-45*, *mir-50*, *mir-71, mir-228) *are shared with *C. elegans*, where all except *mir-50 *are also abundant (using the same read number criteria) [42]. While most of the highly expressed parasite miRNAs are abundant in both the L3 and adult stage, there are a few exceptions, with *mir-2b *and *let-7 *being found only in the adult stage of *B. pahangi *(Figure 2A), while *H. contortus mir-1 *is present only in the L3 stage (Figure 2B). For *B. pahangi *a divergent member of the *let-7 *family, *bpa-mir-5364*, is the most abundant miRNA in adults while a novel sequence, *hco-mir-5983*, is one of the most highly expressed sequences in adult *H. contortus*.

### Temporal expression profiles of miRNAs

**Figure 3:**
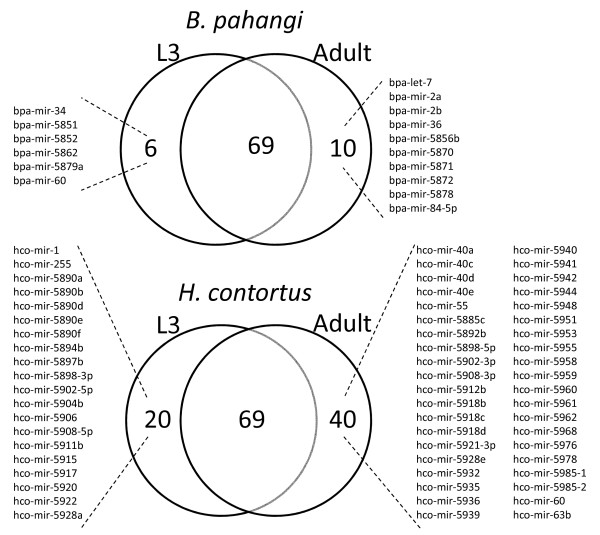
**miRNA temporal expression**. Venn diagram representing the number of miRNAs identified by deep sequencing that were found either in L3 or adult, or in both stages. Stage-specificity was defined as a normalised read count of ≥ 10 in one stage with zero counts in the other stage.

To examine miRNA temporal expression profiles we first identified those for which we found evidence of expression in a single developmental stage only. This was defined as a normalised read count of ≥10 in one stage and no counts in the other stage. Of the 99 unique mature *B. pahangi *miRNAs identified by library sequencing, 14 had reads in one stage only but below this cut-off. Of the remaining miRNAs, six were found only in the L3 stage, 10 in the adult stage only, while 69 were expressed in both stages (Figure 3). The adult-only set for *B. pahangi *contains *let-7*, corresponding to the expression in *C. elegans *where *let-7 *is found at significant levels from mid-L3 onward [42]. For *H. contortus*, of the 180 unique mature miRNA sequences identified, 51 were found in only one stage but fell below the read count threshold. Of the remainder, 20 were found in the L3 stage only, 40 in the adult stage only, and 69 in both stages (Figure 3). We note that as the adult libraries of both parasites were generated from mixed sex worms, these may include miRNAs derived from embryos developing within the females.

**Figure 4:**
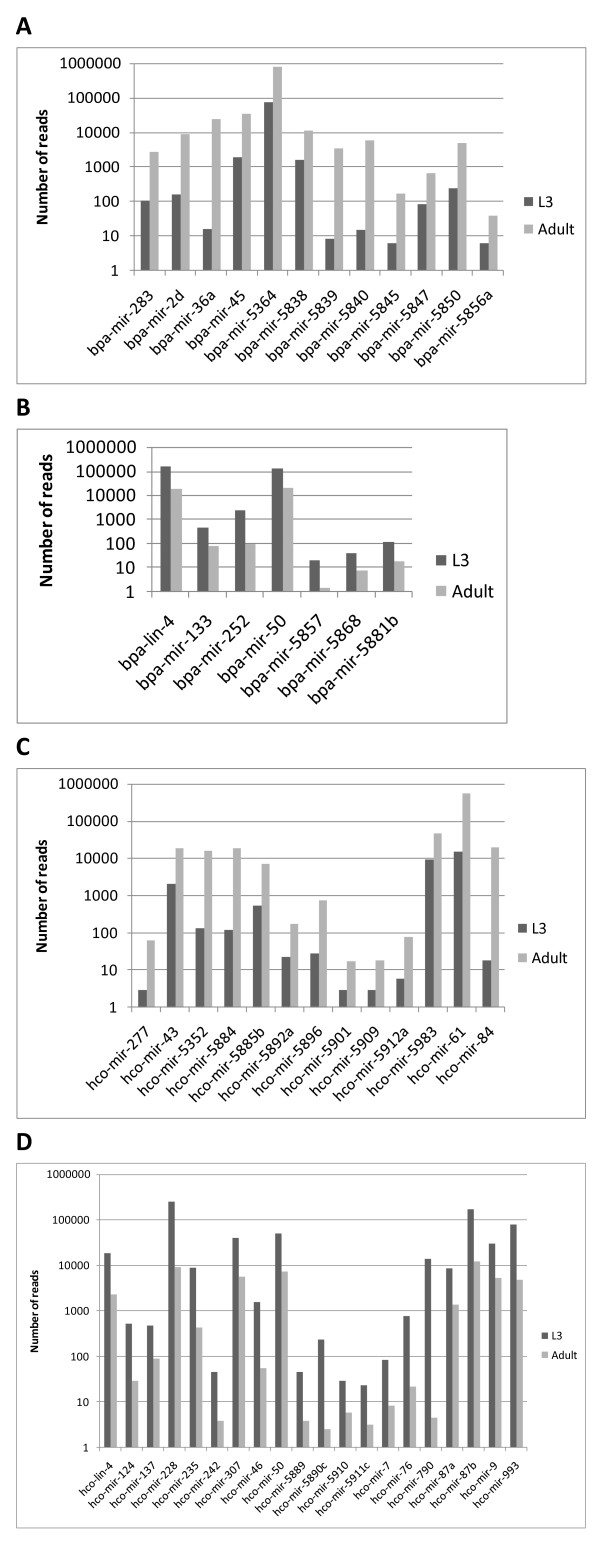
**Differentially expressed miRNAs**. miRNAs that were expressed in both stages but showed > 5-fold changes in abundance between L3 and adult worms. Read numbers are normalised and illustrated on a log scale with the read numbers indicated. A) miRNAs from *B. pahangi *found in the adult stage at five times greater abundance than L3 stage, B) miRNAs from *B. pahangi *found in the L3 at five times greater abundance than adult, C) miRNAs from *H. contortus *found in the adult at five times greater abundance than L3, D) miRNAs from *H. contortus *found in the L3 at five times greater abundance than adult.

Additional miRNAs potentially involved in temporal regulation of gene expression were identified by comparing the abundance of miRNAs found in both the L3 and adult libraries but at > 5-fold greater abundance in one stage than the other. For *B. pahangi*, 12 unique mature sequences were more abundant in the adult compared to L3 stage (Figure 4A), while seven unique mature sequences show the opposite profile (Figure 4B). For *H. contortus*, 13 miRNAs were more abundant in adult (Figure 4C) with 20 being more highly expressed in L3 (Figure 4D). For both *B. pahangi *and *H. contortus*, *lin-4 *was found at higher abundance in L3 of both species compared to adult worms, a profile similar, but not identical, to the *lin-4 *expression profile in *C. elegans*. *Cel-lin-4 *is found at high levels in young adult hermaphrodites but levels have been shown to be affected by both adult age [51] and sex [42]. qRT-PCR was carried out for a selection of developmentally regulated genes and confirmed expression profiles derived from the deep sequencing data (see Additional File 1, Supplemental Results and Figure S3). The high abundance and dynamic developmental expression profiles found for many novel sequences emphasises the likely importance of these potentially species-specific miRNAs in the biology of both parasites.

### Diversity and conservation of nematode miRNAs

Importantly, comparing the full-length mature sequences (using a criteria of > 70% identity) of the *Brugia *and *H. contortus *miRNAs to all miRBase (release 15) entries, demonstrated that the majority were not conserved in other organisms. Using a combination of BLAST and SSEARCH [52], 42 *Brugia *miRNAs out of a total of 104 (40%) unique mature sequences, were conserved in other species based on our criteria. For *H. contortus*, 54 miRNAs out of a total of 192 (28%) unique mature sequences were conserved. The remainder for each species appeared novel. These numbers are comparable to findings from the necromenic nematode *Pristionchus pacificus*, the only non-*Caenorhabditis *nematode with a substantial set of miRNA sequences deposited in miRBase (release 15) where 33/124 (27%) were conserved in any other species [53].

The BLAST analysis was then used to identify the extent of miRNA conservation in other organisms (Additional File 4). Of the parasitic nematode miRNAs with homologues, several are widely conserved in diverse organisms with 20 *Brugia *and 18 *H. contortus *miRNAs being found in at least 20 species. Based on this analysis we find 15 miRNAs (seven *Brugia*, five *H. contortus*, and three shared) that are found only in nematodes and not in any other species. Additionally, re-analysing these using only a seed match to indicate conservation, confirms that at least *mir-36*, *mir-86*, *mir-265 *and *mir-63 *are found only in worms. No miRNAs were found which were shared between *Brugia *and *H. contortus *only and not in any other species. Interestingly, examination of data from *Ascaris suum *[54], recently available in miRBase release 18, revealed several miRNAs identified in our study which are also found in *Ascaris *but currently in no other species. These are *Bpa-mir-5360*, *-5361*, -*5363*, *-5364*, *-5365a*, *-5365b*, and *-5366*, and *Hco-mir-5352*. The greater number of miRNAs conserved between *Brugia *and *Ascaris *is consistent with their classification as clade III nematodes.

**Figure 5:**
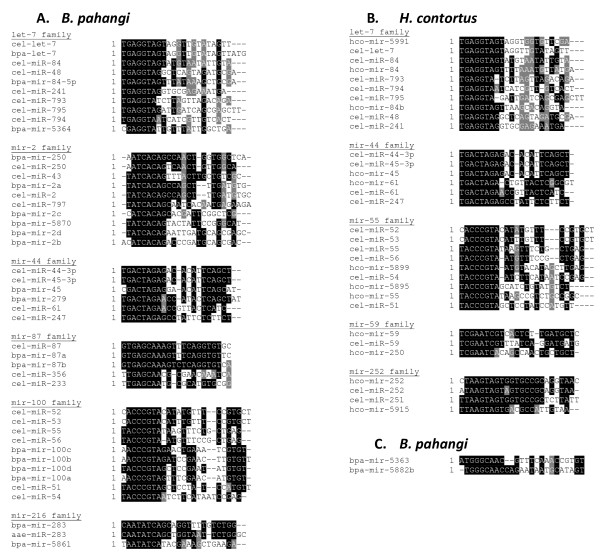
**miRNA families**. Sequence alignments of parasitic nematode miRNAs compared with known miRNAs. Only those families with two or more miRNAs within one parasitic species are shown. A) *B. pahangi *miRNAs compared to *C. elegans *miRNA families. B) *H. contortus *miRNAs compared to *C. elegans *miRNA families. C) A non-conserved *B. pahangi *miRNA family.

A number of miRNAs of *Brugia *and *H. contortus *belong to conserved families, as shown in Figure 5A and 5B. Due to the number of miRNAs discovered, we have only shown those families with two or more miRNAs within one parasitic species. Related sequences were found by assessing nucleotide identity across both the full-length mature miRNA sequence and the seed sequence. Bi-directional miRNAs (described in Supplemental Results, Additional File 1) which were often very similar in sequence, were not included. Figure 5A shows 19 *B. pahangi *sequences compared to sequences from *C. elegans *(where available) falling into six conserved families. For *H. contortus*, the seed match criterion alone was used, which grouped 12 miRNAs into five conserved families (Figure 5B). Many additional *H. contortus *miRNAs showed high identity along their full length (see Supplemental Results in Additional File 1), however, for reasons relating to the current genome assembly (as described in Supplemental Results), some of these may not represent genuine expanded miRNA families. A single case of a novel *B. pahangi *family was found (Figure 5C).

### Conservation of miRNA and host gene organisation

miRNAs can be located intergenically or intragenically. The majority of *C. elegans *miRNAs are located intergenically with only 17% being found within protein-coding genes [48], whereas 40-70% of vertebrate miRNAs are intragenic [48,55]. Examining the genomic context of the parasite miRNAs identified here in relation to annotated genes showed that, like *C. elegans*, the majority were intergenic, but for those that were embedded within a gene (referred to as its host gene) a clear preference for positioning within introns was evident. For *Brugia*, 21 miRNAs were found to be located within protein coding genes (Additional File 5). Four loci (producing three unique mature sequences) were found within exons, with two in the sense and two in the antisense orientation relative to the host gene. 17 *Brugia *miRNAs were intronic, with 15 in the sense and two in the antisense orientation, with the two antisense miRNAs both being part of separate bi-directional pairs, i.e. each has a paired miRNA encoded on the other strand running sense with respect to the host gene. We confirm the previously described [46] locations of *Brugia mir-100a *and *-100d *within an intron and exon, respectively, of the same gene, Bm1_19500 (recombination factor GdRad54, EDP35808), and the location of *Brugia mir-100c *within the intron of Bm1_29425 (nicalin, EDP33842.1).

The intronic-sense context of *Brugia mir-50 *[46] was likewise confirmed. Interestingly, we note that *C. elegans mir-50 *is also located intronically in the sense orientation within an essential gene, Y71G12B.11a, and that the *Brugia mir-50 *host gene, Bm1_56215 (I/LWEQ domain containing protein, EDP28450), is a clear homologue of this gene. Moreover, an *H. contortus mir-50 *homologue was identified and, although classified as intergenic by the preliminary gene predictions used here, BLASTX analysis of ~4 kb of sequence surrounding *hco-mir-50 *against *C. elegans *proteins also provided a match to Y71G12B.11a (positive strand translation). Deeper conservation of this same miRNA/protein-coding gene organisation was also found: the *D. melanogaster rhea *gene (NCBI gene ID: 38978) and human talin 2 (NCBI gene ID: 83660) (which show 47% identity and 67% similarity to each other), both show 38% identity and 57% similarity at the amino acid level to the *C. elegans mir-50 *host gene Y71G12B.11a, and both contain *mir-190 *homologues (both intronic, sense) which are > 70% identical to the nematode *mir-50 *mature sequences. Additionally, the host gene for *hco-mir-7 *shows homology to *D. melanogaster bancal *(NCBI gene ID: 43862) (39% identity and 59% similarity) and to human heterogeneous nuclear ribonucleoprotein K (NCBI gene ID: 3190) (48% identity and 68% similarity), both of which also contain *mir-7*, thus indicating strong selective pressure to maintain this organisation over a large evolutionary distance.

We extended the analysis of genomic context for the *Brugia *data to include protein coding genes close to miRNAs. Although the average intergenic region for *B. malayi *is ~3.8 kb [31] we found 26 intergenic miRNAs positioned within 1 kb of a gene (Additional File 5). Therefore, depending on the accuracy of protein-coding gene predictions, some of these miRNAs could in fact be situated within these genes. 16 miRNAs were in the same orientation as their neighbouring gene, four were located 3' to the gene (with an average of less than 500 nucleotides between gene end and miRNA start) and 12 were located 5' (average of less than 350 intervening nucleotides). The positioning of these miRNAs relative to protein coding genes suggests that they could be transcribed as a single unit, indicating a functional significance of these linkages. In one case a very similar organisation of miRNA and protein coding gene was found to be conserved in *C. elegans*. *Brugia-bantam *is located 299 nucleotides 5' of the gene Bm1_08910 (sense orientation). The homologous *C. elegans *gene, T07D1.2, contains *mir-81 *(intron 5, antisense) and *mir-82 *(intron 1, sense), and the mature sequences for both show only two mismatches to *bpa-bantam*. Therefore, *bpa-bantam *appears to be homologous to *Cel-mir-82 *due to their mature sequence identity, conserved host gene, and similar relative positioning with respect to this gene.

Based on preliminary gene annotations, five *H. contortus *miRNAs were located within predicted protein coding genes, including *mir-7 *described above, all being within introns in the sense orientation. The lower number of miRNAs found within genes may reflect the greater genome size or the preliminary status of current gene annotations. Evolutionary conservation of *mir*-coding gene organisation was again demonstrated by the positioning of *mir-86 *within the homologous host genes in *H. contortus *and *C. elegans *(Y56A3A.7), although not in *Brugia*.

Although our searches of the PicTar, TargetScan and mirWIP target prediction databases provide no indication that *C. elegans mir-50*, *-82 *and *-86 *target their host gene (results not shown), the positional conservation of miRNA and protein coding genes described above suggests that, with the last shared common ancestor of *C. elegans, H. contortus *and *Brugia *being ~350 million years ago [56], there is selective pressure to maintain these genomic contexts.

### Potential mechanisms for generating miRNA diversity

Novel miRNAs can arise by a number of mechanisms [53] including arm switching and gene duplication. We therefore examined whether these processes may contribute to miRNA diversity in *Brugia *and *H. contortus*. We find two examples in *Brugia *and four in *H. contortus *of the major mature product being generated from alternative arms of the precursor (indicated 5p and 3p) in different lifecycle stages, and one example in *H. contortus *of identical read numbers from both arms in the same stage. Two related novel *H. contortus *sequences *hco-mir-5894a *and *hco-mir-5894b *provide an example of alternative arm usage in duplicated sequences as the mature form is derived from different arms of the hairpin in each (Additional File 1, Figure S2). Changes in arm usage at different developmental times and in different tissues have been reported previously and may allow for functional evolution of miRNA loci [57]. In addition, as previous work described a bias towards location of the mature miRNA in the 3' arm of the hairpin for three *Caenorhabditis *species and *Pristionchus pacificus *[53], we determined if this bias was conserved in parasitic nematodes. Although a tendency towards location to the 3' arm was found (74/134 *Brugia *and 121/192 *H. contortus *major miRNA products were located 3', a 1.2-fold and 1.7-fold increase, respectively), this was not as pronounced as described previously where a 2.1-3.3-fold increase was reported [53]. Duplication followed by sequence divergence can give rise to new miRNA sequences and possible examples of this were also found in *Brugia *and *H. contortus *(see Supplemental Results in Additional File 1). In some instances related miRNAs were present in clusters suggesting recent duplication, as detailed below.

### miRNA clusters

**Figure 6:**
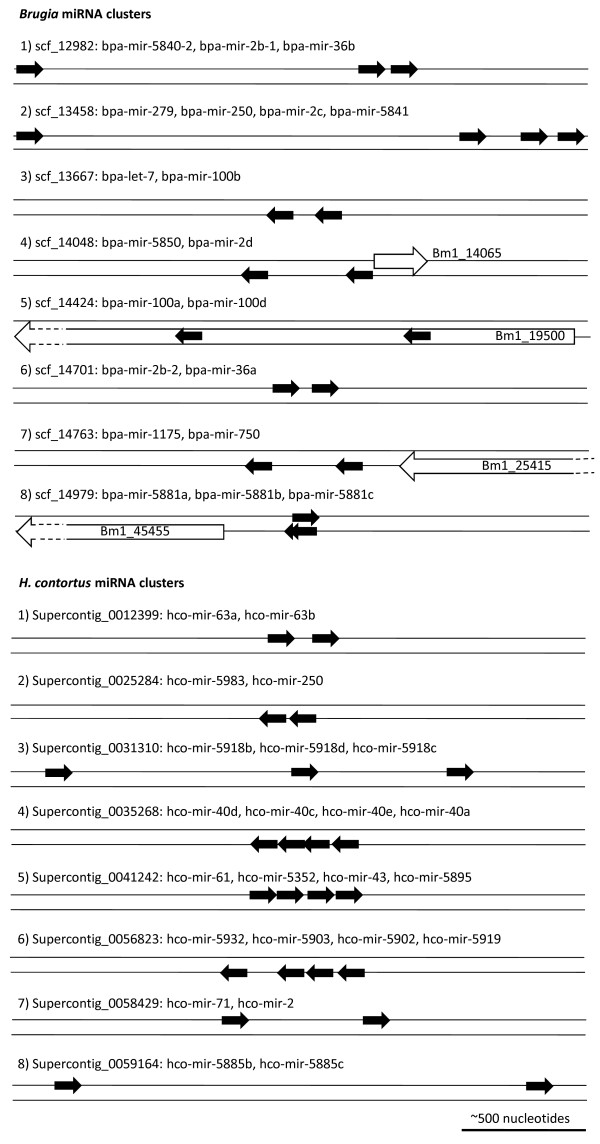
**miRNA clusters**. Schematic representation of identified *Brugia *and *H. contortus *miRNA clusters. Clusters were defined as consecutive miRNAs separated by < 2 kb. Scaffold names and miRNAs contained within the clusters are given. The top line represents the + strand and the bottom line the - strand with the position and direction of each miRNA shown with a black arrow. Protein coding genes containing, or within 1 kb of, a miRNA are represented by an unfilled arrow, with the gene name given.

As miRNAs are often found clustered in the genome [48] we analysed the relative genomic positions of the parasite miRNAs and classified those with a maximum gap of 2 kb between successive miRNAs as clustered (Figure 6 and Additional File 6). This identified eight clusters containing 20 miRNAs for *Brugia*, seven of which contain at least one conserved miRNA. The *Brugia let-7/mir-100b *cluster has been described previously [46], an organisation that is also found in *Drosophila *and humans, but not in *C. elegans *[58]. The *Drosophila *and human *let-7/mir-100 *cluster also contains *mir-125 *which was not identified for *Brugia*. It was not possible to determine if this linkage is conserved in *H. contortus *as no *let-7 *was identified by either deep sequencing or computational discovery approaches, although unmapped reads identical to this miRNA were identified, indicating that *let-7 *is indeed present. The largest cluster identified for *Brugia *contains four miRNAs, three of which are evolutionarily conserved. All are intergenic and transcribed in the same orientation, with *mir-279 *being ~1.6 kb upstream of a 548 nucleotide region containing *mir-250*, *mir-2c *and *bpa-mir-5841 *(Figure 6). This suggests that at least three of these miRNAs are likely to be generated from a single transcript.

Using the same criteria for *H. contortus*, eight clusters consisting of 23 miRNA loci were identified, five of which contain conserved miRNAs (Figure 6). For every cluster all the miRNAs were orientated in the same direction, with three of these consisting of four miRNA loci, each being contained in a genomic region of < 680 nucleotides. Interestingly, *mir-71 *and *mir-2 *are found as a clustered pair in *H. contortus*. Functional roles have been described for *Drosophila mir-2 *in suppressing embryonic apoptosis [59] and for *C. elegans mir-71 *in lifespan and stress responses [60]. Although the genomic organisation of *C. elegans mir-2 *and *mir-71 *(separated by ~7 kb) does not constitute a cluster by the criteria applied here, their relative proximity in *C. elegans *and tight clustering in *H. contortus *may suggest functional linkage of these two miRNAs.

### Endo-siRNA identification from sequencing data

Endo-siRNAs are produced by cells using a pathway very similar to that of miRNAs [1] and have been identified in diverse organisms including *C. elegans *[34]. Endo-siRNAs often target cellular mRNAs, including those derived from transposable elements, to prevent the potentially mutagenic effects of their transposition. We therefore examined the deep sequencing data to identify any small RNAs of this class. Sequences mapping to *B. malayi *coding sequences and *H. contortus *exons in both the sense and antisense orientations were identified using BLASTN, retaining only those with perfect matches over their full length. After removal of sequences corresponding to miRNAs, between 227,319 and 294,245 sense, and between 140,829 and 391,339 antisense reads were identified from the libraries (Table 1). The complete data for the antisense endo-siRNAs for each library is shown in Additional File 7 with the most abundant individual antisense siRNA sequences for each library shown in Additional File 1, Tables S3A-B and S4A-B. The cumulative number of antisense endo-siRNAs matching each coding sequence was determined for *Brugia *with the most targeted genes shown in Additional File 1, Table S3C. Importantly, eight of these were from transposable elements and the total number of sense and antisense sequences matching *Brugia *transposons is shown in Table 3. The same approach was used for *H. contortus *exon sequences and the results are shown in Additional File 1, Table S4C. However, it was not possible to identify siRNA targeting of transposable elements as these annotations are not yet available for *H. contortus*. Alignment of siRNAs to the targeted coding sequences/exons of *Brugia *and *H. contortus *showed clustering of sequences to particular regions of the gene (an example is shown in Additional File 1, Figure S4).

**Table 3:** Sense and antisense transposons reads

Library	Total transposon reads	Sense reads	Antisense reads
***B. pahangi *L3**	30572	9106	21466
***B. pahangi *adult**	71047	34364	36683

Unlike mammals, the *C. elegans *endo-siRNA pathway involves primary endo-siRNAs and secondary molecules generated by RNA-dependent RNA polymerases [1]. These secondary molecules contain 5'-triphosphate residues and, as RNA-dependent RNA polymerases are also present in *B. malayi *[31], it would seem likely that similar secondary molecules will also be produced and that many parasite endo-siRNAs could also contain these modifications. Due to the library preparation procedure used here, the libraries are likely to contain only primary endo-siRNAs. However, the major genes targeted can still be identified (Additional File 1, Tables S3C and S4C) as shown previously [34].

### piRNA identification from sequencing data

piRNAs are critical for germline development in many species and are generated by a distinct mechanism that does not involve Dicer (the biogenesis and functions of piRNAs have been reviewed recently [61]). In *C. elegans*, piRNAs are 21 nucleotides in length, start with a uracil, and possess an upstream sequence motif (GTTTC) [34,62]. These features have been used to identify novel piRNAs in *C. elegans *[42] and *P. pacificus *[53]. We therefore investigated if this process could be extended to *Brugia *and *H. contortus*. The motif was searched for in a defined region upstream from the mapped positions of all 21 U reads which had not been already assigned to another class of RNA. Table 1 shows the total number of reads corresponding to putative piRNAs identified as having an exact match in the upstream motif (full details in Additional File 8). Read numbers were < 50 for both stages of *Brugia *and for the L3 stage of *H. contortus*. However, over 1200 reads, representing 154 loci and 151 unique sequences were identified from the adult *H. contortus *library (Additional File 8). The percentage of loci with the motif in the same upstream location was determined for all 21 U loci and all other read loci (all non-21 U reads) and this is shown for each library in Additional File 1, Table S5. This analysis demonstrates that only in *H. contortus *adults are 21 U loci significantly enriched for the presence of the upstream motif (*p = 0.000*, Chi-square test). In *C. elegans*, piRNAs are most abundantly expressed in young adult worms [42], consistent with their roles in germline development and maintenance. Therefore, as significant numbers of 21 U reads were identified only in the adult *H. contortus *library, and only these loci showed specific enrichment for the presence of the conserved upstream motif, we propose that these represent genuine piRNAs. Their relatively low abundance, in comparison to miRNAs and siRNAs, is in keeping with the results of others [53], and consistent with the libraries being made from whole animal derived material.

## Discussion

Using a combination of deep sequencing and bioinformatic prediction tools we have identified and characterised small RNAs from different lifecycle stages of the parasitic nematodes *Brugia *and *H. contortus*. Comparative analysis with *C. elegans *small RNA data reveals several points of interest.

### The majority of parasitic nematode miRNAs are novel

Many parasite miRNAs identified here were classified as species-specific, based on current sequence data, and did not have homologues in any species, including *Caenorhabditis *spp., which currently represent the most in-depth catalogue of nematode miRNAs. Only 42 of 104 *Brugia *and 54 of 192 *H. contortus *miRNAs were conserved in any other organism. Phylogenetic analysis groups *H. contortus *in the closest clade of parasitic nematodes to *C. elegans*. Despite this close relationship, the miRNA repertoire of *H. contortus *is no more similar to *C. elegans *than more distantly related parasitic species, including *Brugia *(this work) and *Ascaris *[54]. This indicates that the majority of nematode miRNAs are evolutionary diverse and is consistent with comparative studies across the Metazoa showing that most clades contain unique miRNAs. These data suggest that this class of small regulatory RNAs is being continuously expanded [63,64]

The current version of miRBase (release 18) contains 223 *C. elegans *miRNAs, with these data derived from detailed studies of miRNA function in the worm as well as from deep sequencing of multiple stages [42]. Our current analysis accounts only for those miRNAs expressed in L3 and adult worms and a small number of bioinformatically predicted conserved miRNAs. It is probable that additional non-conserved miRNAs are present in the *Brugia *and *H. contortus *genomes and these will be identified by analysis of additional lifecycle stages. Although we have classified a high number of miRNAs from *H. contortus *and *Brugia *as unique, some of these may yet be identified in closely related species. Indeed, several of the miRNAs we describe as currently being found only in *Brugia *and *Ascaris *can be found by homology in the genome databases of other filarial nematodes (unpublished data). As additional data from other parasitic nematode genomes becomes available our results will aid the annotation of miRNAs and advance the understanding of nematode miRNA evolution.

An important proviso of this study was that the *B. pahangi *sequencing reads were, of necessity, mapped to the *B. malayi *genome. Thus any *B. pahangi*-specific sequences will not have been identified. While this explanation may account for the lower number of miRNAs found for *B. pahangi *compared to *H. contortus*, it is unlikely to fully explain the apparent expansion of miRNAs in *H. contortus*. In addition, homologous or heterologous probing of the *B. malayi *V2 gene array with *B. malayi *and *B. pahangi *cDNA showed no obvious bias towards *B. malayi *[65]. An alternative explanation for the difference in number of miRNAs identified may relate to genome sizes for *Brugia *and *H. contortus*. From current sequence data, the *B. malayi *genome is estimated to be 90-95 Mb [31], similar to *C. elegans *(100 Mb). However, the current estimate for *H. contortus *is 300-500 Mb (JSG and MB, unpublished data), a more precise figure awaiting the assembly and annotation of the genome. From studies on *H. contortus *gene structure to date, it appears that most genes are 2-3 times the size of their orthologues in *C. elegans *due to the greater size and number of introns [66]. Whether this expansion in non-coding sequence has resulted in a parallel expansion in small RNAs within the *H. contortus *genome remains speculative for the time being.

Understanding the functional relevance of diversity in miRNA sequences in these parasitic nematodes will require additional study; key to this will be defining their mRNA targets and demonstrating their biological relevance. As small non-coding RNAs provide a mechanism for altering levels of gene expression, their analysis may provide clues as to the evolution of parasitic life styles. The findings of this study provide the basis for further detailed analysis of miRNA expression profiles, both temporal and spatial, and of their target genes, using computational and experimental approaches. Of particular interest will be novel miRNAs that are abundantly expressed, such as *bpa-mir-5364*, and those with stage-restricted expression profiles, which will be relevant to understanding mechanisms of parasite development and responses to host signals.

### Conservation of miRNA-host gene positioning

In the midst of such diversity, comparison of the few evolutionary conserved miRNAs identified in this study demonstrates a remarkable degree of conservation in genomic positioning of certain miRNAs. For example, the observation that *mir-50 *is located within a homologous gene in three different nematode species, argues strongly for the conservation of miRNA function and suggests a strong selective pressure to maintain this genomic positioning. That these partnerships have been maintained over ~350 million years of evolution adds further weight to this hypothesis. This conservation is particularly surprising as there is little conserved microsynteny between these species [31,66,67] and thus may indicate that the function of these miRNAs and their host genes are linked. In some cases, conservation of miRNA and host gene positioning was conserved even in higher eukaryotes, suggesting an ancient origin of such associations.

### Expression of piRNAs in *H. contortus*

A recent study detailing small RNAs from *Ascaris *suggested that the piRNA pathway has been lost from this parasitic nematode [54]. It was speculated that this may reflect adaptation to development at 37°C within a vertebrate host, altering the role of piRNAs in temperature sensitive pathways, such as germline maturation [54]. Our identification of piRNAs in *H. contortus *adult worms, suggests that, at least within clade V parasitic nematodes, the potential roles of piRNAs are conserved. Absolute confirmation of these sequences as piRNAs will require further study to show their association with Piwi proteins in the relevant tissues. However, sequence comparison studies have identified the Piwi related gene, *prg-1*, from *H. contortus *and related clade V parasitic species [68], supporting the presence of this pathway. This provides further evidence of the diversity of small RNAs in nematodes and questions why and when the piRNA pathway has arisen. It is possible that the role of piRNAs has altered in species protected from extreme environmental conditions (the L1-L3 stage of *Ascaris *develops within an egg, those of *Brugia *within a mosquito vector, while the larval stages of *H. contortus *are found free within the environment), but sequence data from more species will help test such speculation.

## Conclusions

Using deep-sequencing and homology-based bioinformatics we have carried out the first in-depth study of small regulatory RNAs in *Brugia *and *H. contortus*, and compared these to *C. elegans*. Our findings demonstrate that many miRNAs in both parasitic species are unique and show little overlap with those of *C. elegans *or any other organism. Some of these novel microRNAs are both abundantly expressed and developmentally regulated. The association of certain miRNAs within host genes is highly conserved across species indicating a functional association. siRNAs and piRNAs were also identified, the latter only in *H. contortus *adult stage, supporting a conserved role of piRNAs in reproduction of some parasitic nematodes. Our results highlight the evolutionary diversity in microRNA repertoire between free-living and pathogenic species, and may provide clues to the evolution of parasitic lifestyles and adaptation to different environments. The availability of genome data for additional parasitic nematode species in the next few years [32] will greatly improve our understanding of novel miRNAs in these organisms, their expansion/loss and potential mRNA targets.

## Methods

### Nematode strains and maintenance

The *B. pahangi *strain used in this study was derived from the original isolate reported by Buckley and Edeson [69]. *Aedes aegypti *mosquitoes (*refm *strain) were infected with *B. pahangi *by membrane feeding on rabbit blood containing first stage larvae (microfilariae). L3 were isolated from mosquitoes after 10 days at 27°C. L3 were used to infect the laboratory host, the jird (*Meriones unguiculatus*) and adult *B. pahangi *worms were isolated after 3-4 months [70]. *H. contortus *worms were a gift from the Moredun Research Institute (Edinburgh) and were the strain MHco3(ISE), which is used for the on-going genome sequencing project. Ensheathed L3 stage larvae were harvested from faecal cultures, while mixed sex adult worms were collected at necropsy (day 28) from Suffolk lambs (5-9 months old) infected with 5,000 L3 larvae and maintained indoors to eliminate risk of other nematode infections. All procedures were carried out in accordance with the Animal (Scientific Procedures) Act, UK.

### Sources of genome assemblies and annotations

The *Brugia malayi *genome assembly [31] (*Brugia*_assembly.ghedin.fasta) was obtained from the Sanger Institute FTP site [71]. *B. malayi *coding sequences were downloaded from NCBI [72] by exporting GenBank coding sequence files. The *H. contortus *genome data (combined_worms_supercontigs200808.fasta) was obtained from the Sanger Institute FTP site [73]. *H. contortus *gene annotations were provided pre-publication from The Wellcome Trust Sanger Institute.

### tRNA and rRNA sequences

The predicted locations of tRNA encoding sequences were identified from the *B. malayi *and *H. contortus *assemblies using tRNAscan-SE [74,75]. Except where noted the default settings were used for all computer algorithms. *Brugia *(*B. malayi *and *B. pahangi*) and *H. contortus *nuclear rRNA sequences were retrieved from the NCBI nucleotide database [76] using the search phrases "*Brugia *rRNA" or "*Haemonchus contortus *rRNA". Additional *Brugia *sequences not already identified were retrieved from the SILVA database [77,78], while the Rfam database [79] provided *Brugia *5S and 5.8S sequences. No further *H. contortus *sequences were identified from either SILVA or Rfam databases. The combined sequences for each organism were compared to a list of *C. elegans *rRNAs derived from WormBase [80] which indicated that the *Brugia *rRNA list contained partial sequences for *B. malayi *28S (AAQA01003089, ~3000 bases, ~600 bases missing at the 5' end), and 18S (AF036588, ~40 bases missing at the 5' end); *B. malayi *and *B. pahangi *sequences for full length 5.8S (with internal transcribed spacer sequences) (EU373625 and EU373655), and *B. malayi *and *B. pahangi *sequences for full length 5S rRNA (with SL1 sequences) (D87037 and D87038), as well as a number of redundant and partial sequences for all forms. The *H. contortus *rRNA list contained full length sequences for 28S (AM039742), 18S (L04153), 5.8S (with internal transcribed spacer sequences) (EU084689) and 5S rRNA sequences (U32122) as well as a number of redundant and partial sequences.

### Small RNA library preparation and sequencing

For both organisms, small RNA libraries were constructed from two developmental stages, infective third stage larvae (L3) and mixed-sex adult worms. Total RNA was extracted by re-suspending frozen nematodes in at least six volumes of TRIzol Reagent (Invitrogen). The material was disrupted using a 3 ml glass hand-held homogeniser (Jencons) and the RNA isolated following the manufacturer's protocol. RNA integrity was determined using an Agilent 2100 Bioanalyzer and RNA Nano kit [81]. For each library, 10 μg of total RNA was treated with 2 units of (RNase-Free) DNase I (Ambion), the reaction stopped by addition of EDTA to 5 mM, and the RNA precipitated by addition of 1/10 volume 3 M NaCl, 2.5 volumes of 100% ethanol at -80°C. A Small RNA Sample Prep kit (Illumina) was used to prepare the libraries, as described in Supplemental Methods in Additional File 1. Libraries were analysed with an Agilent 2100 Bioanalyzer and DNA 1000 kit to determine size and molarity. DNA was diluted to 4 nM for direct sequencing using an Illumina Cluster Station and 3G Genome Analyzer following the manufacturer's protocols.

### Small RNA library sequence processing and miRNA identification

Two algorithms were employed to identify miRNAs from the small RNA library sequences data, miRDeep [43,82], and MIREAP [44,45,83] both of which also require RNAfold (part of the Vienna RNA package) [50,84]. Importantly, scoring by these programs takes into account that reads should map in a manner consistent with the cellular processes by which miRNAs are generated. Therefore the relative positions of putative mature and star strands within the hairpin are assessed on their similarity to that expected from the products of Drosha/Dicer processing. Illumina sequence reads were processed for input to these programs and mapped to the genome as detailed in Supplemental Methods in Additional File 1. miRDeep has been shown to perform equally well either with data filtered to remove sequences representing regions of annotated function or on unfiltered data [43]. Unfiltered data was therefore used with both programs and miRNA predictions that overlapped with other classes of non-coding RNA subsequently removed. The prediction outputs of miRDeep and MIREAP were processed to remove any with minimum free energies (MFE) > -25 kcal/mol. The prediction sets produced from both programs were merged, then further processed to identify identical or near identical sequences mapping to different genomic locations and to find predictions common to both the L3 and adult libraries. These candidate precursors sequences were then analysed using BLASTN [49] against sets of tRNA and rRNA (produced as described above) and any with > 20 bases of > 93% identity removed. *Brugia *precursors were also analysed by BLASTN against transposable element genomic sequences (derived from co-ordinates given in the supporting online material of [31]) and genomic repeat element sequences (accession numbers, M12691.1, M12692.1 and M34369.1). A final high confidence set of miRNAs was then generated by i) retaining all predictions supported by at least one read corresponding to the star strand ii) predictions where no star strand was identified were retained only if the mature sequence was supported by 10 or more reads (combined from both lifecycle stages) and the precursor sequence passed the hairpin classifier CIDmiRNA [47,85]. This algorithm was developed as a tool for *ab inito *miRNA identification in the human genome and has also been employed as a filtering step for miRNA identification from deep sequencing [86,87]. It was verified as suitable for application to the parasitic nematode data by first testing it using worm (nematode and platyhelmith) precursor sequences from miRBase release 15 [88]. From 788 sequences, three with ambiguous bases were removed and the program identified 683 of the remaining 785 (87%) as miRNA hairpins (for the test sequences Window Length lower value was set to 50 to account for the short length of some sequences). For analysis of miRNA abundance levels read counts were normalised to the total number of reads mapping for each library.

### miRNA prediction by homology

All miRNA mature and star sequences reported for the following worm species: *C. briggsae *(cbr), *C. elegans *(cel), *C. remanei *(crm), *P. pacificus *(ppc), *S. japonicum *(sja), *S. mansoni *(sma) and *Schmidtea mediterranea *(sme), were downloaded from miRBase [48] release 15 [88]. These sequences were then used to query the *B. malayi *and *H. contortus *genome assemblies using the BLASTN algorithm with parameters adjusted to account for the short query length (word size set to 7 and e-value threshold to 85). All BLAST hits were then extended to match the length and positioning of the query sequence to give hits of ~22 bases representing candidate mature sequences. For each of these, two candidate precursors were generated by extracting genomic sequences corresponding to i) 70 nucleotides upstream and 10 nucleotides downstream and, ii) 10 nucleotides upstream and 70 nucleotides downstream, with respect to the ~22 nucleotide region. Candidates were then filtered using RNAfold, CIDmiRNA and manual scoring as detailed in Supplemental Methods in Additional File 1.

### miRNA families and conservation

miRNAs were identified as homologous to known miRNAs present in miRBase 15 using BLASTN (word size 7, e-value 85) to compare full-length mature sequences, allowing only sense matches and applying a 70% nucleotide identity cut-off. This process was used to assign gene names for conserved miRNA identified by deep-sequencing and to determine inter-species conservation of miRNAs. To provide additional homology assignments for gene naming, mature sequences were searched against sequences using SSEARCH [52] at miRBase (e-value cutoff 200) and homology assigned if a seed match and at least 16 nucleotides of identity over an 18 base region were present. All final miRNA names were assigned by miRBase.

To identify related miRNAs within each species, the complete sets of full-length mature sequences were examined using ClustalX. In addition, to identify sequences related by seed sequence (identical 5' bases 1-7 or 2-8) identity over the first 10 bases was examined. The full-length sequences for miRNAs identified using either criteria were then aligned using ClustalX and Boxshade alongside *C. elegans *miRNA family members where available (*C. elegans *families defined essentially as described previously [89]).

### miRNA genomic context

For *B. pahangi*, all miRNAs were classified as exonic, intronic or intergenic (with protein coding genes within 1 kb noted) by analysing *B. malayi *genomic scaffold GenBank annotation files via NCBI Batch Entrez (accession numbers for miRNA scaffolds in Additional File 2) using the Vector NTi sequence analysis suite (Invitrogen). For *H. contortus*, genomic positioning was determined by BLAST analysis on sequence sets extracted using the annotated genome file data. For *C. elegans *and other species data on genomic context and miRNA clustering was derived from miRBase [48].

### Identification of siRNAs and piRNAs from deep sequencing data

siRNAs were identified by matching library sequences to the coding sequences (*Brugia*) or exons (*H. contortus*) using BLASTN. Only sequences with perfect identity along the full length of the read sequence were retained and those already identified as miRNAs excluded. A custom Perl script was used to map the retained sequences to coding sequences/exons. To identify putative piRNAs, library sequences of 21 nucleotides in length starting with a uracil were assessed. 21 U sequences that had been previously annotated as miRNA, coding sequence/exons, rRNA or tRNA were removed (for *Brugia *21 U reads that mapped to transposons were not excluded due to the potential overlap in these classes) and a custom Perl script used to extract 80 nucleotides of upstream genomic sequence. The presence of a consensus motif, GTTTC, between positions -48 and -42 of the upstream region was then determined [34,42,53].

## Authors' contributions

ADW performed the molecular biology, bioinformatic analysis, and participated in the design and coordination of the study. WW and MH performed the bioinformatic analysis. MB performed the deep sequencing. MB and JSG carried out the genome annotation for *H. contortus*. ED and CB conceived of the study and participated in its design and coordination. ADW, ED and CB wrote the manuscript. All authors read and approved the final manuscript.

## Supplementary Material

Additional file 1 **Supplemental Results, Methods, Figures and Tables**. This file contains supplemental results, supplemental methods, four supplemental figures (Figures S1-S4), nine supplemental tables (Tables S1, S2, S3A-S3C, S4A-S4C, and S5), and supplemental references. Click here for file

Additional file 2 **miRNAs identified by deep sequencing**. This file contains full details of the miRNAs identified by deep sequencing. Click here for file

Additional file 3 **miRNAs predicted by homology**. This file contains full details of the miRNAs predicted by homology. Click here for file

Additional file 4 **Evolutionary conservation of miRNAs**. This file contains the analysis of the evolutionary conservation of the identified miRNAs. Click here for file

Additional file 5 **miRNAs associated with protein coding genes**. This file describes miRNAs associated with protein coding genes. Click here for file

Additional file 6 **miRNA clusters**. This file describes miRNA found clustered in the genome. Click here for file

Additional file 7 **siRNAs**. This file contains anti-sense sequences for all libraries which were identified as endo-siRNAs. Click here for file

Additional file 8 **piRNAs**. This file contains sequences identified from the piRNA analysis. Click here for file
