# Supplementary material for: Diversity in parasitic nematode genomes: the microRNAs of Brugia pahangi and Haemonchus contortus are largely novel
Source: BMC Genomics. 2012 Jan 4;13:4. doi: 10.1186/1471-2164-13-4 (PMC3282659; doi:10.1186/1471-2164-13-4)
Supplement: Additional file 1 — Supplemental Results, Methods, Figures and Tables. This file contains supplemental results, supplemental methods, four supplemental figures (Figures S1-S4), nine supplemental tables (Tables S1, S2, S3A-S3C, S4A-S4C, and S5), and supplemental references. [file 1471-2164-13-4-S1.PDF]

Additional File 1

Supplemental Results, Methods, Figures and Tables

This file contains supplemental results, supplemental methods, four supplemental figures (Figures S1-S4), nine supplemental tables (Tables S1, S2, S3A-S3C, S4A-S4C, and S5) and supplemental references.

SUPPLEMENTAL RESULTS

Reporting of a single genomic locus per unique *H. contortus* mature miRNA

Due to the advanced assembly of the *B. malayi* genome, all genomic locations for *Brugia* sequences were reported as these may represent genuine multiple loci. However, for *H. contortus,* the identification program outputs contained a large number of cases where either i) multiple identical precursors mapped to different loci, or, ii) multiple similar precursors, with identical mature sequences, mapped to multiple loci. The *H. contortus* genome is only partially assembled and contains a large amount of sequence heterogeneity. Therefore, although in some cases multiple loci could reflect the real genomic situation, it was not possible to determine when this was the case. The number of multiple loci and multiple similar sequences was therefore simplified by reporting a single precursor sequence and genomic locus for each unique mature sequence. The following criteria were used when selecting a supercontig location for a particular miRNA;

Supercontig locations were preferentially selected if they:

i) contained other clustered miRNAs

ii) were supported by mapping of a star strand

iii) represented one of a majority of identical sequences in situations where some identical and some similar sequences were reported

iv) contained the sequence with the lowest free energy value for the miRNA hairpin

v) were the location reported by miRDeep

Similar *H. contortus* mature miRNA sequences

The process described above was applied in order to remove the reporting of multiple precursors representing the same *H. contortus* miRNA. However, for the remaining unique mature miRNAs it was possible that sequence heterogeneity in the mature region could result in the same miRNA being reported more than once. To assess this possibility, and determine if this could contribute to the greater number of miRNAs identified in *H. contortus* compared to *B. pahangi,* all the mature miRNA sequences were aligned using ClustalX and those showing similarity along their full length are presented along with their precursors (Additional File 1, Figure S2).

Evidence for additional miRNAs in the unmapped sequence data

As the reference genome sequences for both parasites are currently incomplete it was possible that some of the unmapped reads could represent additional miRNAs. To investigate this possibility the unmapped sequence data was analysed for mature miRNA sequences that are highly conserved across species but which were not identified from the mapped data. This highlighted reads with perfect homology to *mir-1* (*B. pahangi*) and *let-7* (*H. contortus*) in the unmapped data which would not have been identified previously as the flanking sequences were not available, precluding analysis of the folded precursor structure. Although this approach provided evidence for the presence of additional miRNAs we do not report these in our analysis and it was not performed on a larger scale as analysis of the folded precursor was considered essential for confident miRNA annotation.

miRNA duplications and bidirectional loci

For *Brugia* a number of miRNA have duplications in the genome with identical or highly similar sequences being found on different genomic scaffolds, although confirmation of these as true duplicates will depend upon future refinement of the genome assembly. In two

cases *bpa-mir-5847* and *bpa-mir-5873* there are duplications, both between scaffolds and within a scaffold. For *H. contortus* potential duplications on different supercontigs have not been analysed due to the preliminary nature of the genome assembly (as described above). However, loci present on the same supercontig producing related mature sequences are often found as clusters; three of the four clustered miRNAs on supercontig 0035268 have related mature sequences, two clustered on supercontig 0059164 are related (a third related sequence is on the same supercontig ~6kb distant), and a clustered pair on supercontig 0012399 are both homologous to *mir-63* (Additional File 1, Figure S2). Three *Brugia* clusters contain miRNA related in sequence, the *mir-100a/mir-100d* cluster [1], a cluster containing *mir-2c* and *mir-250* (see Figure 6, main text), and a cluster on scaffold 14979.

Bi-directional loci result from expression of miRNA precursors from both the sense and antisense strand at the same genomic locus. These have been described in detail in *Drosophila* and are also found in mammals [2-4]. Expression from bi-directional loci was found for novel miRNA genes in both *Brugia* (8 pairs, producing 12 unique mature sequences; *bpa-mir-5856a* and *b*, *bpa-mir-5879a* and *b*, *bpa-mir-5880a* and *b*, *bpa-mir-5881a* and *b*, *bpa-mir-5882a* and *b*, *bpa-mir-5365a* and *b*), and *H. contortus*, (2 pairs giving rise to 4 unique mature miRNA; *hco-mir-5984a* and *b*, *hco-mir-5985-1* and *-2*). Some of these pairs give related mature sequences as found for *bpa-mir-5880a* and *b*, *bpa-mir-5881a* and *b*, and both *H. contortus* pairs.

Confirmation of miRNA differential expression patterns by qRT-PCR

qRT-PCR was used to confirm the expression profiles of a number of miRNAs identified as being temporally regulated from the deep sequencing data. These were randomly selected from the sets described in Figures 4A-D (main text). For deep sequencing, mixed sex adult

material was used, however, due to their greater size, the majority of RNA was likely to have derived from females. The expression profiles of five *B. pahangi* and five *H. contortus* miRNAs were therefore examined in L3 and adult females by qRT-PCR (Additional File 1, Figure S3A and B). For each species, results were normalised to miRNAs (*bpa-mir-100c* and *hco-mir-5899*) found to be consistently expressed throughout the lifecycle from microarray analysis (result not shown). All miRNA tested showed a similar temporal profile to that found by deep sequencing.

SUPPLEMENTAL METHODS

Small RNA library preparation

A Small RNA Sample Prep kit (Illumina) was used to prepare libraries for deep sequencing. The additional reagents required along with this kit were; Novex 15% TBE-Urea polyacrylamide gels (Invitrogen), T4 RNA ligase 2 (truncated) (NEB), SuperScript II Reverse Transcriptase (Invitrogen), Novex 6% TBE polyacrylamide gels (Invitrogen). For each library 10 μg of DNase I-treated total RNA was size fractionated on a denaturing 15% TBE-urea polyacrylamide gel and the section corresponding to ~15-30 nucleotides purified as described in the Illumina protocol (Preparing Samples for Analysis of Small RNA). All subsequent RNA manipulations were performed using Non-Stick RNase-free microfuge tubes (Ambion). Thereafter libraries were prepared essentially following the Alternative v1.5 Protocol (Illumina), also modified slightly as noted below. The main steps performed were ligation of a pre-adenylated 3′ adaptor to the population of small RNA molecules using T4 RNA ligase 2 (truncated) enzyme (NEB), followed by ligation of a 5′ adapter using T4 RNA ligase (both ligation incubation times extended to 2 hours). The adaptor-ligated RNA was reverse transcribed to single stranded cDNA using SuperScript II Reverse Transcriptase and a primer complementary to the 3′ adapter sequence. The cDNA was PCR amplified with Hotstart Phusion DNA polymerase (NEB) for 14 cycles using primers binding to the regions

generated via the adapters. The resulting population of cDNAs were fractionated on a 6% TBE polyacrylamide gel and a band of 90-100 bp excised and purified. In order to limit possible bias in the population of molecules generated, and also to incorporate a negative control, the RT-PCR step was adjusted so that for each library all the adaptor-ligated RNA was used in four separate reactions, three reverse transcriptase containing reactions (RT+) and one reaction with no enzyme. All the cDNA was used in multiple independent PCR reactions and all the RT+ amplified material was pooled.

Small RNA library sequence processing

Illumina sequence reads were processed using a custom Python script to identify identical reads and convert these to unique sequences with associated read counts. NCBI blastall version 2.2.15 (settings -p blastn -e 1e-10 -F F) was used to remove reads matching adapter sequences only. Sequences were mapped to the genome using MicroRazerS version 0.1. [5] and the output converted for input to MIREAP (version 0.2) using a custom Python script. For miRDeep the input sequence files prepared for MIREAP were further processed to clip 3′ adapter sequence and identify duplicates. Sequences were mapped to the genome using NCBI megablast version 2.2.15 (settings -W 12 -D 2 -p 100), retaining only perfect alignments, and miRDeep run with two adjustments, the optional randfold scoring was not used and settings -a 3 -c 5 were used for the script filter_alignments.pl.

miRNA prediction by homology

The ~102 base candidate precursors extracted from the genome were examined using RNAfold [6, 7] and those with a minimum free energy of ≤ -25 kcal/mol retained. Duplicated sequences and precursors containing ambiguous nucleotides were removed. The remainder were examined using CIDmiRNA [8, 9] and retained only if passed by this program, with the additional requirement that the hairpin reported (60-100 nucleotide) contained >85% of the

predicted mature sequence. Candidate sequences (original ~102 nucleotide versions) were then refolded using the mfold Quikfold server [10] (default settings for Energy Rules RNA 3.0 except Maximum Number of Foldings restricted to 1) [11, 12]. The structures were then manually scored to determine if they met the following criteria; i) mature sequence contained on one arm of the hairpin ii) 14 bases of the mature sequence involved in base pairing with the other arm iii) a four base maximum bulge size in the stem. For candidates that passed these criteria the mature region was used in a SSEARCH [13] against mature miRNA sequences at miRBase [14]. Sequences were retained if they contained 16 nucleotides of identity over an 18 base region and/or an exact match over the seed region (5′ bases 1-7 or 2-8). Those already identified by deep-sequencing were removed. Candidate sequences were examined on a microarray (results to be presented elsewhere), and the final homology based predictions retained if they satisfied either of the following criteria; i) fulfilled both the seed match and overall length homology, ii) produced a signal over a threshold value on the majority of microarray chips and fulfilled at least one of the homology criteria.

miRNA qRT-PCR

DNAse I treated total RNA isolated from L3 and adult females of *B. pahangi* and *H. contortus* (see main text for methods) was polyadenylated and reverse transcribed following the miRNA 1st-Strand cDNA Synthesis protocol (Agilent Technologies). qPCR was performed following the miRNA qPCR Master Mix protocol (Agilent Technologies) using an Agilent Mx3005P qPCR System. Sequences of the oligonucleotides used are given in Additional File 1, Table S2**.** All cDNA synthesis reactions were performed using three biological replicates and all qPCR reactions were carried out in triplicate.

SUPPLEMENTAL FIGURES

Figure S1. miRNA length

Size distribution of *B. pahangi* and *H. contortus* mature miRNAs identified by deep sequencing.

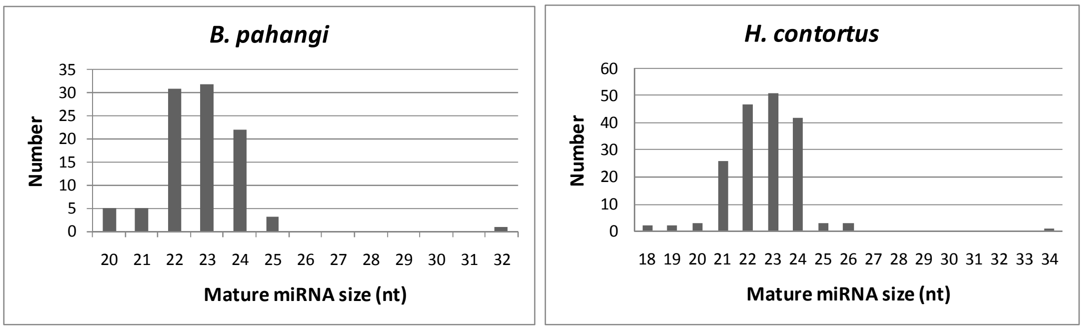

Figure S2. Alignments of similar *H. contortus* miRNAs

The relationship between similar mature *H. contortus* miRNAs was examined by aligning mature and precursor sequences in ClustalX.

```
hco-mir-63a    1------AATGACACTGTTGCGAACTGGGAT
hco-mir-63b    1-----CTATGACACTGTCGCGAATTGGGG-

hco-mir-63a    1CTTGTATTCTTCTGATGATCATACCTGTTCGACGGGAGTCATCGTCCTTAT--GGTACAA
hco-mir-63b    1-----------CTCGTCCTCACTCCGATTCGCCGGTAGTCATCGTCATCGGTGAGCACTA

hco-mir-63a    59TGACACTGTTGCGAACTGGGATGGTCAAAGGCATTCGTTCCGCATA
hco-mir-63b    50TGACACTGTCGCGAATTGGGGTGATTAGAGC---------------
```

```
hco-mir-87a    1------GTGAGCAAAGTTTCAGGTGTGC
hco-mir-87b    1------GTGAGCAAAGTCTCAGGTGTGG

hco-mir-87a    1----CCATGAATGAGCTCCGTCTCGCCGCCTGAACCTTTCGTCTCAACCTCTATGCCGGG
hco-mir-87b    1TACCCTATAAGGATGGTCCATC---CCGCCTGA--CACTTGACTCAAACCTCATTTAAAG

hco-mir-87a    57CAGGTGAGCAAAGTTTCAGGTGTG-CGAGCGGACTCGTATACTCCGTTGCC
hco-mir-87b    56AAGGTGAGCAAAGTCTCAGGTGTGGCGGCCTATCCTAGATNNNNNNNNNNG
```

```
hco-mir-40b    1--------TCACCGGGTGTCTTGTAGCGGA
hco-mir-40d    1--------TCACCGGGTATCTTGCAGCGGG
hco-mir-40a    1--------TCACCGGGTGTCTTGCAGTGAG
hco-mir-40c    1--------TCACCGGGAGTTCTGTGGTGA-

hco-mir-40d    1GTACGCTACTGTATCTGGCGTTCGTTGTAGGACC-CCCTG-TGAGGCTTTATGGGACTTC
hco-mir-40a    1-------ACCGGG---AAAACTCACTGTAAGACCATCCAG-TAAAGCATCGCAAGACTTC
hco-mir-40b    1GTCGGCCGCATCTCCAGCGGTCCGCTACAATACCGTCGT--TGAAGCGTTCATGGTCTTC
hco-mir-40c    1-CAACACCTAGCGAGATGGAATCACCTCAGAATAACCTTGGTGACGCTGATTAAGACTTC

hco-mir-40d    59ACCGGGTATCTTGCAGCGGGTGCTTGGACAGACAGTATGGCTC
hco-mir-40a    50ACCGGGTGTCTTGCAGTGAGATTTTAGAAT-------------
hco-mir-40b    59ACCGGGTGTCTTGTAGCGGACATGCTGAGCGGTTATACCACTG
hco-mir-40c    60ACCGGGAGTTCTGTGGTGA-TACCTACTTTATTCCTCCAAAC-
```

```
hco-mir-5886a  1----------GAACAATCTTTGACTTTGGTT---
hco-mir-5886b  1----------GAACACTCTTTGACTTTGGTTTGA


hco-mir-5886a  1ATGGAACCCCTATTATATTGCATGTTCTTCAAACGGAGTCATCGATGTGTTCTTGTACAA
hco-mir-5886b  1--------CTTTTTATATTGCATTTTGTTCAAACTGAGTCATTGATGTGTTCTTGTTCAA

hco-mir-5886a  61TGCTAAGAACAATCTTTGACTTTGGTTTGAAGAAATATATGCAGTCTGGA--------
hco-mir-5886b  53TACTAAGAACACTCTTTGACTTTGGTTTGAAGAATTATATGCAATCAGATCCTAGAAT
```

```
hco-mir-5892a  1----------AATTAACATTTGGATCATCTGAA-
hco-mir-5892b  1----------AATTAACATTTGGATCATCTGAGC


hco-mir-5892a  1TAACGAGCCTTCCGGTCCTGAAGTTTGCTCAGATGATCCAGATGTTAGTCGCCCGCATGG
hco-mir-5892b  1---CGAGCCTTCCGGTCCTGAAGTTTGCTCAGATGATCCAGATGTTAGTCGCCCGCATGG

hco-mir-5892a  61AAAGCAATTAACATTTGGATCATCTGAACGATCTTCGAGGATGCTATTCG----------
hco-mir-5892b  58AAAGCAATTAACATTTGGATCATCTGAGCGATATTCGAGGATGCTATTCGAAGCCTGTAC

hco-mir-5892a     ---------------------
hco-mir-5892b  118TCCTTATTCCATCTACTGCAC
```

```
hco-mir-5885a  1----------TGAGATCACGCGTATATTCGC--
hco-mir-5885c  1----------TGAGATCACGCGTATATTCGCTA
hco-mir-5885b  1----------TGAGATCACGCCTATATTCGC--


hco-mir-5885b  1----ATCGTGAAACGGTCCGTACGCCAGGGTATGGGTGGTGGTCTGGTAAGCATGACGCT
hco-mir-5885c  1GTCCGCCAATAAACGGCTGAT-TATCAGAGTATACGTGGTGTTCTGGTAAGCATGACGCT
hco-mir-5885a  1----GCGCTCGGTCGGCACCGATAACAGGGTGTACGTGGTGGTCTGGTAAGTGTGTCGCT

hco-mir-5885b  57TATGAGATCACGCCTATATTCGCTTTGTACAAGTTCGATTCCTGCCCAA----
hco-mir-5885c  60TATGAGATCACGCGTATATTCGCT--AGACAAGTTCGAATCCTCGAGGGGAGT
hco-mir-5885a  57TATGAGATCACGCGTATATTCGCC--GCGCAAGTTCGACGCCTGTCGACG---
```

```
hco-mir-5984a  1--------GTGTACTCTTTAGACGGTTCTA
hco-mir-5984b  1--------GCGTACTCTTTAGACGGTTT--


hco-mir-5984a  1ACCGAATATTTTGGCCATCATAAAACCGTCTAAAGAGTACGCTTCTATGCATAGAAGTGT
hco-mir-5984b  1------------AATCATCATAGAACCGTCTAAAGAGTACACTTCTATGCATAGAAGCGT

hco-mir-5984a  61ACTCTTTAGACGGTTCTATGATGATTTTTATACCTTCTTGC
hco-mir-5984b  49ACTCTTTAGACGGTTTTATGATGGCC---------------
```

```
hco-mir-5890a  1----------TACCCCTTTTCATTTTTATGC
hco-mir-5890d  1---------CTACCCCTTTCCATTCTTATGC


hco-mir-5890a  1------AGTCACGCATTTATGCGGCAGCATAGGACCGAATTGTGGGAGAACTTCTATGAT
hco-mir-5890d  1ACTCCTAGTCACGCATTTATGCGGCAGCACAGGACTGAACTGTGGGAGGACTTATATGAT

hco-mir-5890a  55TCTACCCCTTTTCATTTTTATGCTGCTCTGGAGGTGGATTCACAACTTTATCGCTA
hco-mir-5890d  61TCTACCCCTTTCCATTCTTATGCTGCTTTGGAG-----------------------
```

```
hco-mir-5897b  1-----------TTTTGTATGCCTTATCTGGAAT-
hco-mir-5897c  1-----------TTTTGTATGTATTATCTGGAATT


hco-mir-5897b  1CACTATAATTTGCTTGGGTTGGAGATTCAGTCCCAGATAATGCGTACAAAGTCCGACTAT
hco-mir-5897c  1---------------TGGTTGGAAGAACAGTCCCAGATGATTCGTACAAAGTCCGACCAT

hco-mir-5897b  61AGGAGGTTTTGTATGCCTTATCTGGAATTGATCACAACCTTTATGCAGAA
hco-mir-5897c  46AGGAGGTTTTGTATGTATTATCTGGAATTGTGCACAACC-----------
```

```
hco-mir-5894a  1----------CCTATATCTG-ATGGTGTAGCGGGA
hco-mir-5894b  1---------GCCTATATCTG-ATGGTGTAGC----
```

```
hco-mir-5894a  1CAGAAAAAGTAAGTGTGGTAAGCCTATATCTGATGGTGTAGCGGGAATTCTCGTCTCGCT
hco-mir-5894b  1------------------------TATTTTTACTGTTGCTTATCAGATTATAGGTTAGCT
```

```
hco-mir-5894a  61ACACCATCAGTTATGGACTTGGCTACCTTATAACTAATGTG
hco-mir-5894b  37ATGCCTATATCTGATGGTGTAGCGGAAACGAGA--------
```

Note-mature sequences on different arms:
hco-mir-5894a
cagaaaaagtaagtgtggtaagcCCTATATCTGATGGTGTAGCGGGAattctcgtctcgctacaccatcagttatggacttggctac
cttataactaatgtg
hco-mir-5894b
tatttttactgttgcttatcagattataggttagctatGCCTATATCTGATGGTGTAGCggaaacgaga

---

```
hco-mir-5928a  1---------TGAAGTAGAGTAGATTTGTAGGATC
hco-mir-5928e  1--------TTCAAGTAAAGTAGGTTAGTAGGATC
hco-mir-5928b  1---------TGAAGTAAGGTAGATTAGTAAGGT-
hco-mir-5928c  1---------TGAAGTAAGGTAGACTAGTAAGGT-
hco-mir-5928d  1---------TGAAGTAGAATAGTTGGGTATGGT-
```

```
hco-mir-5928d  1-------------GGCACAAACCTGAAGTAGAATAGTTGGGTATGGTCAGCTGATCCTAC
hco-mir-5928a  1-GCCTATCATTTGGGCCCAAATTTGAAGTAGAGTAGATTTGTAGGATCAGTTGACCCTAC
hco-mir-5928b  1-------------GGCCCAAAACTGAAGTAAGGTAGATTAGTAAGGTCAGCTGATCCTAC
hco-mir-5928c  1-------------GGCCCAATTCTGAAGTAAGGTAGACTAGTAAGGTCAGTTGATCCTAC
hco-mir-5928e  1ATAGGATCATTGGGGCACAGATTTCAAGTAAAGTAGGTTAGTAGGATCAGCTGACTCTAC
```

```
hco-mir-5928d  48TGATCTACTCTGCTTCAAGTTTGTGCTC------------------------------
hco-mir-5928a  60TGAACCGCTCTACTTCGAGTTTGTGCCCCGAAAATTGGCGTCATAGGCGTA--------
hco-mir-5928b  48TATTCTACTGTACTTCGAAATTGGGCCCAA----------------------------
hco-mir-5928c  48TATTCTACTGTACTTCGAAATCGGGCCCAA----------------------------
hco-mir-5928e  61TAAACTATCTTACTTCGAATTTGTGCCCCAATGATAGGCGTTAGAGTCGT--------
```

```
hco-mir-5890e  1----------ATAGGACTGAACTGTGGGAGA-
hco-mir-5890f  1----------ATAGGACTGAACTGTGGGAGGA
hco-mir-5890b  1---------CATAGGACTGAATTGTGGGAG--
hco-mir-5890c  1---------CATATAACTGAACTGTGGGAG--
```

```
hco-mir-5890c  1-----------TATGCGGCAGCATATAACTGAACTGTGGGAGGACTCATATGATTCTACC
hco-mir-5890e  1AGTCACGCATTTATGCGGCAGCATAGGACTGAACTGTGGGAGAACTTATATGATTCTACC
hco-mir-5890b  1-----------TATGCGGCAGCATAGGACTGAATTGTGGGAGGACTTATATGATCCTACC
hco-mir-5890f  1---------TTTATGCGGCAGCATAGGACTGAACTGTGGGAGGACTAATGTGATTCTACC
```

```
hco-mir-5890c  50CCTCTTCATTTTTATGCTGCTTCGGAG--------------------------------
hco-mir-5890e  61CCTTTTCATACTTATGCTGCTTCAGAGGTGAATTCACAA--------------------
hco-mir-5890b  50CCTTTTCATTTTTATGCTGCTTTGGAG--------------------------------
hco-mir-5890f  52CCTTTCCATTCTTATGCTGCTTTGGAGGTGAATTCACAACTTTCCCGCTAGTATCCACC
```

```
hco-mir-5911b  1------TAAGGTTGACTT---GCTTAGAAGCT
hco-mir-5911c  1------TGAGGCTGACTT---GCTTAGGAGCT
```

```
hco-mir-5911b  1GAGGGTGTTGTAAGGTTGACTTGCTTAGAAGCTTGTTTATTCTTCTAAGCTCTTCACTCT
hco-mir-5911c  1AATGATGTTGTGAGGCTGACTTGCTTAGGAGCTTGTTCGTGTTCCTAAGCTCTTCACCCT
```

```
hco-mir-5911b  61TATAGACACCTTCA--------
hco-mir-5911c  61TACAGACACCTTCAGCATTAGA
```

```
hco-mir-5985-1 1AAACACTGACTTGTAACATCGGC-
hco-mir-5985-2 1-AACACTGACTTGTAACATCGGCT
```

```
hco-mir-5985-1 1GCGAGGATGAAAACACTGACTTGTAACATCGGCTGGCCACCAGCCGATGTTACAAGTCAG
hco-mir-5985-2 1-CGAGGATAAAAACACTGACTTGTAACATCGGCTGGTGGCCAGCCGATGTTACAAGTCAG
```

```
hco-mir-5985-1 61TGTTTTTATCCTCGCA
hco-mir-5985-2 60TGTTTTCATCCTCGC-
```

Figure S3. miRNA qRT-PCR

qRT-PCR using comparative quantification of miRNA expression in L3 relative to adult female. A) *B. pahangi* miRNAs expression in mosquito derived L3 relative to adult female, B) *H. contortus* miRNAs expression in ensheathed L3 relative to adult female.

A

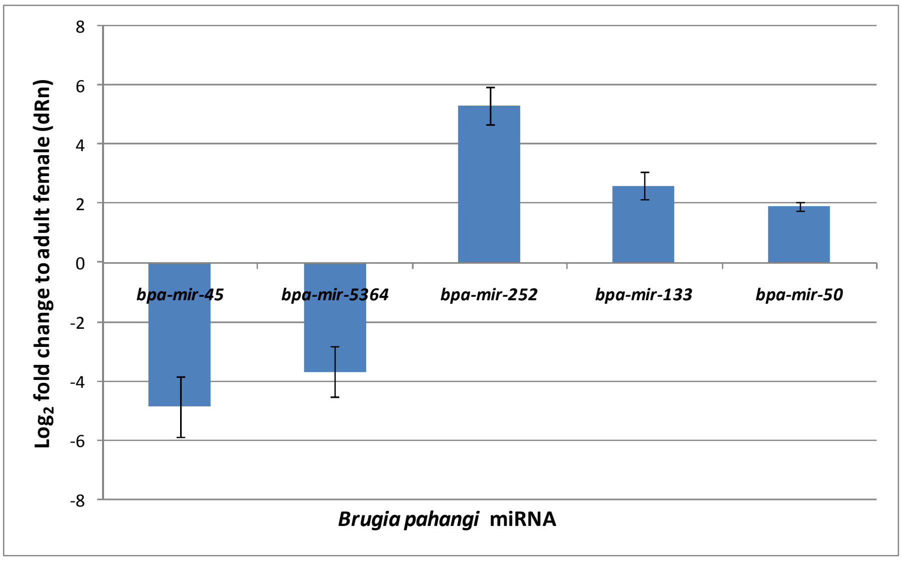

B

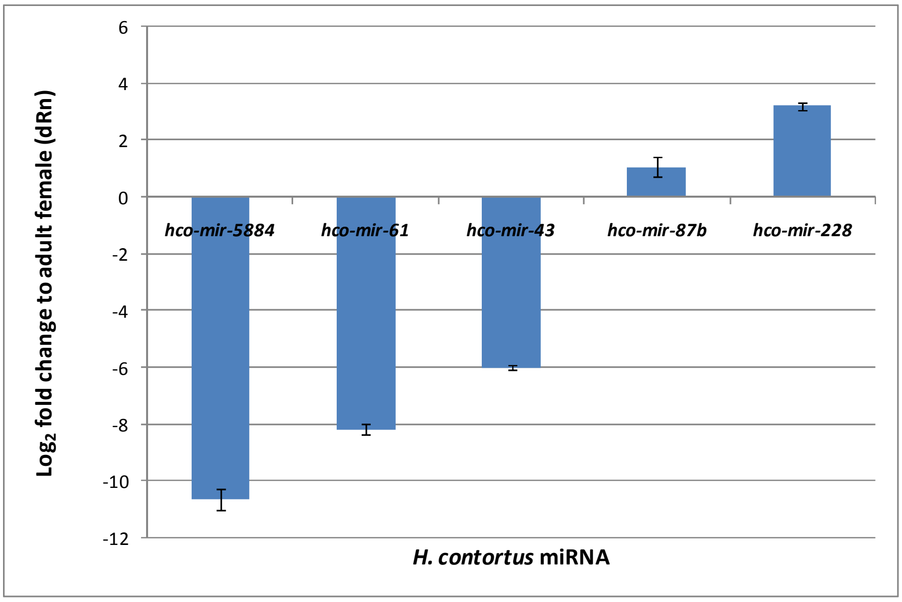

Figure S4. siRNAs aligned to *Brugia* gene Bm1_56425.

The coding sequence for Bm1_56425 is indicated on the first line of the alignment with sense (indicated 5′) and antisense (indicated 3′) siRNAs aligned beneath. Unique sequence identifiers contain details of the library from which they were found (identifiers beginning 3978_3 are from *B. pahangi* L3 and 3984_3 from *B. pahangi* adult). The number of reads found for each unique sequence is given in the next column. The alignment is presented showing only sequences represented by at least three sense reads and at least 10 antisense reads.

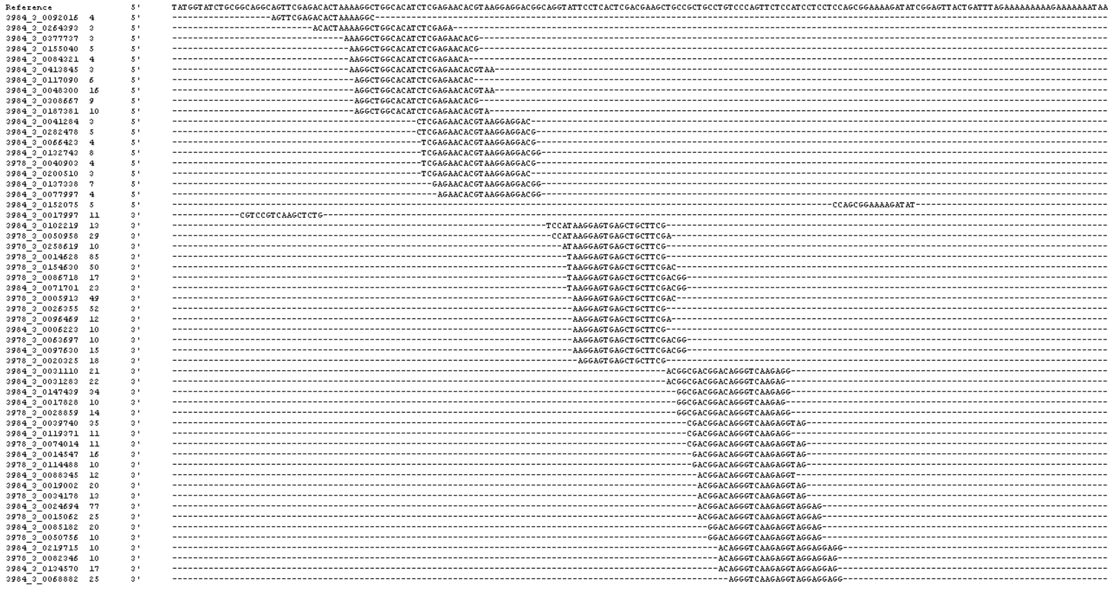

SUPPLEMENTAL TABLES

Table S1. Program identification

Total sequences	miRDeep and MIREAP	miRDeep only	MIREAP only
125 *B. pahangi*	50	31	44
180 *H. contortus*	77	48	55

Table S2. qRT-PCR oligonucleotide sequences

Name	Sequence, 5′-3′	Description
oADW081	ACTAGAGGAACATTCAGCAT (20)	bpa-mir-45 forward primer for qRT-PCR.
oADW082	CGAGGTATTGTTTATTGGCT (20)	bpa-mir-5364 forward primer for qRT-PCR.
oADW083	CTAAGTAGTAGTGCCGCA (18)	bpa-mir-252 forward primer for qRT-PCR.
oADW084	ATTGGTCCCCTTCAACCA (18)	bpa-mir-133 forward primer for qRT-PCR.
oADW085	TGATATGTCTGATATTCTTGG (21)	bpa-mir-50 forward primer for qRT-PCR.
oADW092	AACCCGTAGAACTGAAATC (19)	bpa-mir-100c forward primer for qRT-PCR. Normalising miRNA.
oADW086	TAGGGTACTGACATTGAATGA (21)	hco-mir-5884 forward primer for qRT-PCR
oADW087	TAGACTGTTACTCGGCGT (18)	hco-mir-61 forward primer for qRT-PCR
oADW088	TATCACAGTGTCATTGGGT (19)	hco-mir-43 forward primer for qRT-PCR
oADW089	CAAAGTCTCAGGTGTGG (17)	hco-mir-87b forward primer for qRT-PCR
oADW091	AATGGCACTGCATGAATTCA (20)	hco-mir-228 forward primer for qRT-PCR
oADW096	TACCCGTAATGTACATAGC (19)	hco-mir-5899 forward primer for qRT-PCR. Normalising miRNA.

Table S3A. Most abundant individual antisense endo-siRNAs for *Brugia* L3. Non-normalised read numbers are shown.

Sequence	Length and 1st nucleotide	Reads	Coding sequence
TGAATTAACAGATATGTTTGCAT	23T	9128	Bm1_47680
GTGCGAGACCCGGGTTCGATTCCCGGCCGGGGAG	34G	5999	Bm1_10415
GGCGAGATGGCCGAGCGGTCTAAGGC	26G	4073	Bm1_53495
GGCGAGATGGCCGAGCGGTCTAAGG	25G	2222	Bm1_53495
ATATGGAAATGTAGCGTATAGGTATAA	27A	1786	Bm1_01155
ATATGGAAATGTAGCGTATAGGTATA	26A	1502	Bm1_01155
ATGGAAATGTAGCGTATAGGTATA	24A	1386	Bm1_01155
ATGGAAATGTAGCGTATA	18A	1270	Bm1_01155
ATGGAAATGTAGCGTATAGGTATAA	25A	1216	Bm1_01155
TCGGGTTGTTTGAGATTGCA	20T	948	Bm1_12630
CGGGTTGTTTGAGATTGCA	19C	860	Bm1_12630
TTGAATTAACAGATATGTTTGCAT	24T	855	Bm1_47680
TATGGAAATGTAGCGTATAGGTATA	25T	841	Bm1_01155
TCCTCGGTAGTATAGTGGTGAGTATCCGCGC	31T	737	Bm1_10415
GTGGATCACTTGGCTCATGGATC	23G	731	Bm1_28700
ATGGAAATGTAGCGTATAGGTAT	23A	676	Bm1_01155
TATGGAAATGTAGCGTATA	19T	675	Bm1_01155
ATGGAAATGTAGCGTATAGG	20A	656	Bm1_01155
TGGAAATGTAGCGTATAGGTAT	22T	616	Bm1_01155
CGGGTTGTTTGAGATTGCAGC	21C	605	Bm1_12630

Table S3B. Most abundant individual antisense endo-siRNAs for *Brugia* adult. Non-normalised read numbers are shown.

Sequence	Length and 1st nucleotide	Reads	Coding sequence
GTTGCATGCAATAGGGCGGAGGTAGA	26G	14488	Bm1_23695
GGCGAGATGGCCGAGCGGTCTAAGG	25G	14138	Bm1_53495
ATATGGAAATGTAGCGTATAGGTATAA	27A	12192	Bm1_01155
ATATGGAAATGTAGCGTATAGGTATA	26A	9076	Bm1_01155
ATGGAAATGTAGCGTATAGGTATAA	25A	8738	Bm1_01155
ATGGAAATGTAGCGTATAGGTATA	24A	7591	Bm1_01155
GGCGAGATGGCCGAGCGGTCTAAGGC	26G	6396	Bm1_53495
GTAGTATAGTGGTGAGTATCCGCG	24G	6294	Bm1_10415
AGGAGTGGAGCCTGCGGCTTAAT	23A	4925	Bm1_28695
ATGGAAATGTAGCGTATA	18A	4177	Bm1_01155
ATGGAAATGTAGCGTATAGGTATAACTATC	30A	3707	Bm1_01155
TGGACCGTAGCGAGACGTACGA	22T	3198	Bm1_28690
AGGAACGGACTCCCTGGAATC	21A	3150	Bm1_28705
TATGGAAATGTAGCGTATAGGTATA	25T	3056	Bm1_01155
TGAATTAACAGATATGTTTGCAT	23T	3019	Bm1_47680
ATATGGAAATGTAGCGTATA	20A	2809	Bm1_01155
TGGCTCATGGATCGATGAA	19T	2530	Bm1_28700
TCCTCGGTAGTATAGTGGTGAGTATCCGCG	30T	2433	Bm1_10415
TCCTCGGTAGTATAGTGG	18T	2385	Bm1_10415
TATGGAAATGTAGCGTATAGGTATAACTATC	31T	2315	Bm1_01155

Table S3C. Highest siRNA-targeted *Brugia* coding sequences

Coding sequence	L3 reads	Adult reads	Description	Protein ID	
Bm1_01155	28730	40064	hypothetical protein	EDP39449.1	
Bm1_12630	37504	7981	hypothetical protein	EDP37167.1	
Bm1_10415	11610	19183	hypothetical protein	EDP37611.1	
Bm1_28700	14401	13866	hypothetical protein	EDP33958.1	
Bm1_53495	9434	13382	hypothetical protein	EDP28985.1	
Bm1_47680	10593	2570	conserved hypothetical protein	EDP29677.1	Transposon
Bm1_23695	*1*	9733	hypothetical protein	EDP34957.1	
Bm1_28705	1633	7314	rRNA promoter binding protein, putative	EDP33959.1	
Bm1_28690	*0*	7449	hypothetical protein	EDP33956.1	
Bm1_01405	*0*	6741	rRNA promoter binding protein, putative	EDP39400.1	
Bm1_28695	*0*	6063	hypothetical protein	EDP33957.1	
Bm1_44325	*0*	3770	Senescence-associated protein, putative	EDP31077.1	
Bm1_05565	1829	1752	hypothetical protein	EDP38583.1	
Bm1_29195	1086	2488	hypothetical protein	EDP33869.1	Transposon
Bm1_40115	1598	*1184*	hypothetical protein	EDP32286.1	Transposon
Bm1_11205	1024	1348	Zinc knuckle family protein	EDP37454.1	Transposon
Bm1_13675	*251*[a]	2090	hypothetical protein	EDP36963.1	
Bm1_36940	1035	1228	Pyridine nucleotide-disulphide oxidoreductase, dimerisation domain containing protein	EDP31642.1	
Bm1_29340	697	1376	gag protein, putative	EDP33826.1	Transposon
Bm1_13465	1086	*736*	gag protein, putative	EDP37003.1	Transposon
Bm1_11025	*0*	1523	hypothetical protein	EDP37487.1	
Bm1_49085	410	*886*	Myb-like DNA-binding domain containing protein	EDP29961.1	
Bm1_25970	*0*	1244	hypothetical protein	EDP34501.1	
Bm1_56425	716	*347*	hypothetical protein	EDP28408.1	
Bm1_40120	521	*519*	conserved hypothetical protein	EDP32287.1	Transposon
Bm1_39410	531	*442*	Pao retrotransposon peptidase family protein	EDP32144.1	Transposon
Bm1_09785	397	*69*	proprotein convertase aPC6C isoform, putative	EDP37735.1	
Bm1_51430	448	*17*	Choline/Carnitine o-acyltransferase family protein	EDP29313.1	

The 20 coding sequences with the highest cumulative siRNAs matches from each stage were identified and combined. Normalised read numbers are given.

[a] The italicised figures indicate that siRNAs for that coding sequence were not in the most abundant set in this stage.

Table S4A. Most abundant individual antisense endo-siRNAs for *H. contortus* L3. Non-normalised read numbers are shown.

Sequence	Length and 1st nucleotide	Reads	Exon	Gene
GTTGAGAAATGTAGCGTATAGGTAT	25G	8781	exon_Supercontig_0031379_starts_at_883	HCON02341
GTTGAGAAATGTAGCGTATAGG	22G	7165	exon_Supercontig_0031379_starts_at_883	HCON02341
TGAGAAATGTAGCGTATAGGTAT	23T	6404	exon_Supercontig_0031379_starts_at_883	HCON02341
CAGATCACTCTGGTTCAATGTCGGG	25C	4562	exon_Supercontig_0029517_starts_at_806	HCON02531
TGAGAAATGTAGCGTATAGG	20T	4193	exon_Supercontig_0031379_starts_at_883	HCON02341
TTGAGAAATGTAGCGTATAGGTAT	24T	3383	exon_Supercontig_0031379_starts_at_883	HCON02341
GTTGAGAAATGTAGCGTA	18G	3275	exon_Supercontig_0031379_starts_at_883	HCON02341
CAGATCACTCTGGTTCAATGTCGG	24C	2988	exon_Supercontig_0029517_starts_at_806	HCON02531
GTTGAGAAATGTAGCGTATA	20G	2933	exon_Supercontig_0031379_starts_at_883	HCON02341
TTGAGAAATGTAGCGTATAGG	21T	2359	exon_Supercontig_0031379_starts_at_883	HCON02341
TTGAGAAATGTAGCGTA	17T	2183	exon_Supercontig_0031379_starts_at_883	HCON02341
TACCTGATTGATTCTGTCAGC	21T	1867	exon_Supercontig_0007912_starts_at_5260	HCON00911
CCGTTGAGAAATGTAGCGTATAGGTAT	27C	1833	exon_Supercontig_0031379_starts_at_883	HCON02341
AGGTGTTGTATCCAGTAGAGCA	22A	1796	exon_Supercontig_0029517_starts_at_806	HCON02531
CAGATCACTCTGGTTCAATGTCGGGG	26C	1785	exon_Supercontig_0029517_starts_at_806	HCON02531
TGAGAAATGTAGCGTA	16T	1678	exon_Supercontig_0031379_starts_at_883	HCON02341
AAAGCCAGATCACTCTGGTTCAATGTCGGGG	31A	1568	exon_Supercontig_0029517_starts_at_806	HCON02531
CACCGTTGAGAAATGTAGCGTATAGGTAT	29C	1522	exon_Supercontig_0031379_starts_at_883	HCON02341
ACCGTTGAGAAATGTAGCGTATAGGTAT	28A	1511	exon_Supercontig_0031379_starts_at_883	HCON02341
AGATCACTCTGGTTCAATGTCGGG	24A	1319	exon_Supercontig_0029517_starts_at_806	HCON02531

Table S4B. Most abundant individual antisense endo-siRNAs for *H. contortus* adult. Non-normalised read numbers are shown.

Sequence	Length and 1st nucleotide	Reads	Exon	Gene
ACTGCGATCTGTTGAGACTATCCTTTGATCGGGT	34A	6117	exon_Supercontig_0029517_starts_at_806	HCON02531
ACTGCGATCTGTTGAGACTATCCTTTGATCGGG	33A	5263	exon_Supercontig_0029517_starts_at_806	HCON02531
TACCTGATTGATTCTGTCAGCGC	23T	3722	exon_Supercontig_0007912_starts_at_5260	HCON00911
TACTGCGATCTGTTGAGACTATCCTTTGATCGGG	34T	3658	exon_Supercontig_0029517_starts_at_806	HCON02531
AATTAGAGTGCTCAGAACAA	20A	3428	exon_Supercontig_0038837_starts_at_21	HCON02728/ HCON02729
AAATTAGAGTGCTCAGAACAA	21A	3017	exon_Supercontig_0038837_starts_at_21	HCON02728/ HCON02729
TACCTGATTGATTCTGTCAGC	21T	2637	exon_Supercontig_0007912_starts_at_5260	HCON00911
ATTAGAGTGCTCAGAACAA	19A	2217	exon_Supercontig_0038837_starts_at_21	HCON02728/ HCON02729
GATCACTCTGGTTCAATGTCGG	22G	1840	exon_Supercontig_0029517_starts_at_806	HCON02531
TACTGCGATCTGTTGAGACTATCCTTTGATCGGGT	35T	1333	exon_Supercontig_0029517_starts_at_806	HCON02531
AGATCACTCTGGTTCAATGTCGGG	24A	1323	exon_Supercontig_0029517_starts_at_806	HCON02531
GTTGAGACTATCCTTTGAT	19G	1285	exon_Supercontig_0029517_starts_at_806	HCON02531
GCTCGTAGTTGGATCT	16G	1216	exon_Supercontig_0038837_starts_at_21	HCON02728/ HCON02729
AGATCACTCTGGTTCAATGTCGGGGC	26A	1134	exon_Supercontig_0029517_starts_at_806	HCON02531
GATCACTCTGGTTCAATGTCGGG	23G	1115	exon_Supercontig_0029517_starts_at_806	HCON02531
ATAAATTAGAGTGCTCAGAACAA	23A	1109	exon_Supercontig_0038837_starts_at_21	HCON02728/ HCON02729
GATCACTCTGGTTCAATGTCGGGGC	25G	1105	exon_Supercontig_0029517_starts_at_806	HCON02531
GAATAAATTAGAGTGCTCAGAACAA	25G	1013	exon_Supercontig_0038837_starts_at_21	HCON02728/ HCON02729
TAGCTTCAGCGATGGATCGGTTG	23T	1008	exon_Supercontig_0009638_starts_at_804	HCON05468
GGAGGAAAAGAAACTAAC	18G	1002	exon_Supercontig_0009638_starts_at_377	HCON05467

Table S4C. Highest siRNA-targeted *H. contortus* exons

Exon	L3 reads	Adult reads	Gene
Supercontig 0038837: 21[a]	87812	51585	HCON02728/HCON02729[1]
Supercontig 0029517: 806	63950	43127	HCON02531[2]
Supercontig 0031379: 883	69075	4050	HCON02341[3]
Supercontig 0031379: 1711	18867	25532	HCON02341[3]
Supercontig 0007912: 5260	13420	15239	HCON00911[4]
Supercontig 0038837: 1960	10448	10967	HCON02728/HCON02729[1]
Supercontig 0009638: 377	6249	7926	HCON05467[5]
Supercontig 0007912: 5638	4842	3839	HCON00911[4]
Supercontig 0009638: 804	3236	3777	HCON05468
Supercontig 0008882: 1165	4029	2511	HCON03144
Supercontig 0009638: 772	547	343	HCON05467[5]
Supercontig 0038837: 1778	420	205	HCON02728/HCON02729[1]
Supercontig 0053263: 12581	*5*	232	HCON06036
Supercontig 0058863: 42322	49	167	HCON03609
Supercontig 0029517: 21	119	68	HCON02531[2]
Supercontig 0034878: 17894	*1*	163	HCON01816
Supercontig 0059025: 9477	129	*3*	HCON00799
Supercontig 0004597: 256	*3*	127	HCON01573
Supercontig 0048661: 4151	112	*0*	HCON05425
Supercontig 0057951: 6325	*0*	95	HCON02487
Supercontig 0057954: 11942	*2*	69	HCON06166
Supercontig 0056612: 4132	*0*	68	HCON03183
Supercontig 0059103: 76761	30	*11*	HCON00830
Supercontig 0001976: 1265	12	*26*	HCON00667
Supercontig 0053783: 22025	12	*15*	HCON03236
Supercontig 0047769: 8028	18	*3*	HCON03755

[a] Supercontig and start co-ordinate of exon.

The 20 exons with the highest cumulative siRNAs matches from each stage were identified and combined. The italicised figures indicate that siRNAs for that exon were not in the most abundant set in this stage. Normalised reads numbers are given. Superscript numbers above gene names indicate where multiple exons from the same gene were identified.

Table S5.

	Total loci	Number of times motif found	Percentage frequency of motif	P value for 21U enrichment	piRNA FDR
B. pahangi L3 21U	4945	24	0.48	0.041	64 %
B. pahangi L3 non-21U	273038	872	0.32		
B. pahangi adult 21U	8775	30	0.34	NA	NA
B. pahangi adult non-21U	434183	1576	0.36		
H. contortus L3 21U	6370	20	0.31	0.791	91 %
H. contortus L3 non-21U	396542	1173	0.30		
H. contortus adult 21U	11807	160	1.35	0.000	34 %
H. contortus adult non-21U	922733	4248	0.46		

SUPPLEMENTAL REFERENCES

1. Poole CB, Davis PJ, Jin JM, McReynolds LA: **Cloning and bioinformatic identification of small RNAs in the filarial nematode, *Brugia malayi***. *Molecular and Biochemical Parasitology* 2010, **169**:87-94.
2. Bender W: **MicroRNAs in the *Drosophila* bithorax complex**. *Genes Dev* 2008, **22**:14-19.
3. Stark A, Bushati N, Jan CH, Kheradpour P, Hodges E, Brennecke J, Bartel DP, Cohen SM, Kellis M: **A single Hox locus in *Drosophila* produces functional microRNAs from opposite DNA strands**. *Genes Dev* 2008, **22**:8-13.
4. Tyler DM, Okamura K, Chung WJ, Hagen JW, Berezikov E, Hannon GJ, Lai EC: **Functionally distinct regulatory RNAs generated by bidirectional transcription and processing of microRNA loci**. *Genes Dev* 2008, **22**:26-36.
5. Emde AK, Grunert M, Weese D, Reinert K, Sperling SR: **MicroRazerS: rapid alignment of small RNA reads**. *Bioinformatics* 2010, **26**:123-124.
6. Hofacker IL, Fontana W, Stadler PF, Bonhoeffer LS, Tacker M, Schuster P: **Fast folding and comparison of RNA secondary structures**. *Monatshefte Fur Chemie* 1994, **125**:167-188.
7. **Vienna RNA Package**. [http://www.tbi.univie.ac.at/~ivo/RNA/].
8. Tyagi S, Vaz C, Gupta V, Bhatia R, Maheshwari S, Srinivasan A, Bhattacharya A: **CID-miRNA: A web server for prediction of novel miRNA precursors in human genome**. *Biochem Biophys Res Commun* 2008, **372**:831-834.
9. **CIDmiRNA**. [http://mirna.jnu.ac.in/cidmirna].
10. **The DINAMelt Web Server**. [http://mfold.rna.albany.edu/?q=DINAMelt/Quickfold].
11. Mathews DH, Sabina J, Zuker M, Turner DH: **Expanded sequence dependence of thermodynamic parameters improves prediction of RNA secondary structure**. *J Mol Biol* 1999, **288**:911-940.
12. Zuker M: **Mfold web server for nucleic acid folding and hybridization prediction**. *Nucleic Acids Res* 2003, **31**:3406-3415.
13. Pearson WR: **Searching protein-sequence libraries: comparison of the sensitivity and selectivity of the Smith-Waterman and FASTA algorithms**. *Genomics* 1991, **11**:635-650.
14. **miRBase SSEARCH**. [http://www.mirbase.org/search.shtml].
